# Non‐genetic and genetic rewiring underlie adaptation to hypomorphic alleles of an essential gene

**DOI:** 10.15252/embj.2021107839

**Published:** 2021-09-15

**Authors:** Altea Targa, Katherine E Larrimore, Cheng Kit Wong, Yu Lin Chong, Ronald Fung, Joseph Lee, Hyungwon Choi, Giulia Rancati

**Affiliations:** ^1^ Institute of Medical Biology (IMB) Agency for Science, Technology and Research (A*STAR) Singapore Singapore; ^2^ Skin Research Institute of Singapore (SRIS) Agency for Science, Technology and Research (A*STAR) Singapore Singapore; ^3^ School of Biological Sciences Nanyang Technological University Singapore Singapore; ^4^ Department of Medicine Yong Loo Lin School of Medicine NUS and National University Health System Singapore Singapore

**Keywords:** CRISPR‐Cas9, genetic adaptation, hypomorphic alleles, NPC, transcriptome rewiring, Chromatin, Transcription & Genomics, Evolution & Ecology

## Abstract

Adaptive evolution to cellular stress is a process implicated in a wide range of biological and clinical phenomena. Two major routes of adaptation have been identified: non‐genetic changes, which allow expression of different phenotypes in novel environments, and genetic variation achieved by selection of fitter phenotypes. While these processes are broadly accepted, their temporal and epistatic features in the context of cellular evolution and emerging drug resistance are contentious. In this manuscript, we generated hypomorphic alleles of the essential nuclear pore complex (NPC) gene *NUP58*. By dissecting early and long‐term mechanisms of adaptation in independent clones, we observed that early physiological adaptation correlated with transcriptome rewiring and upregulation of genes known to interact with the NPC; long‐term adaptation and fitness recovery instead occurred *via* focal amplification of *NUP58* and restoration of mutant protein expression. These data support the concept that early phenotypic plasticity allows later acquisition of genetic adaptations to a specific impairment. We propose this approach as a genetic model to mimic targeted drug therapy in human cells and to dissect mechanisms of adaptation.

## Graphical abstract

Generation of hypomorphic alleles of *NUP58* in human cells using CRISPR‐Cas9 provides a proof‐of‐principle demonstration that early non‐genetic changes allow later acquisition of genetic adaptations to a specific impairment.

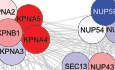

## Introduction

Evolutionary adaptation to adverse conditions is a pervasive trait that enables cells to restore fitness by acquiring compensatory mutations (Bloom & Arnold, 2009). This fundamental process has far‐ranging implications for multiple fields extending from evolutionary biology to human health, including the development of cancer and emergence of drug‐resistant traits (Rokyta *et al*, 2002; Byrne *et al*, 2014; Russo *et al*, 2019). While it is now widely accepted that single‐cell organisms such as yeast and bacteria can rapidly adapt to selective pressure (Moore *et al*, 2000; Blank *et al*, 2014; Szamecz *et al*, 2014), we still lack a clear understanding of the processes that drive this remarkable ability. For instance, while several long‐term evolution experiments have identified specific genetic mechanisms of adaptation (Lang & Desai, 2014), we are currently debating if cellular plasticity predates and facilitates the acquisition of adaptive mutations. Indeed, while it has been argued that cellular plasticity might hinder the acquisition of genetic changes by triggering a fitness gain, recent data suggest that the same cancer type can acquire drug resistance to treatment by a combination of cellular plasticity and genetic adaptation (Shaffer *et al*, 2017; Kim *et al*, 2018; Salgia & Kulkarni, 2018; Bell & Gilan, 2020). However, it remains unclear how genetic and non‐genetic processes interact to facilitate the emergence of drug resistance.

Deleterious polymorphisms are not uncommon, with hypomorphic alleles accounting for up to 12% of the yeast coding genome (Doniger *et al*, 2008) and a range of different mutations being associated with developmental syndromes in humans (Happle, 2003; Arauz *et al*, 2010; Jacobs *et al*, 2011). Moreover, recent large‐scale human genomics analyses revealed the presence of a very small percentage of healthy individuals carrying deleterious genetic mutations (MacArthur & Tyler‐Smith, 2010; MacArthur *et al*, 2012; Chen *et al*, 2016), suggesting compensatory mechanisms exist to compensate the genetic mutation. To date, adaptive evolution in response to hypomorphic mutations has been largely studied in single‐celled organisms, where a wealth of genome editing tools are available. However, recent developments in genome editing techniques using CRISPR‐Cas9 have now eased the generation of hypomorphic alleles in mouse cells (Challa *et al*, 2016). Here, for the first time, we *in vitro* dissected early and late adaptation to the impairment of an essential gene in human cells. We used CRISPR‐Cas9 to edit key regions of the essential nuclear pore complex (NPC) protein NUP58, which was previously shown to be bypassable in yeast (Liu *et al*, 2015), and generated several independent single cell‐derived human clones containing diverse hypomorphic alleles. These mutant NUP proteins were low in abundance and carried large truncations. We observed that all the isolated clones initially upregulated components that were functionally related to the NPC, whereas long‐term gain of fitness correlated with focal amplification of mutant *NUP58*. We speculate that early phenotypic plasticity provides time for cells to survive the initial insult, opening the possibility of genetic adaptations to be acquired and fitness regained. Given that targeted therapy fails to achieve full target inhibition, we propose this approach as an *in vitro* genetic method to dissect early and late mechanisms of drug resistance. This might be relevant for therapies that are already on the market as identification and inhibition of early non‐genetic changes may limit subsequent emergence of drug‐resistant traits.

## Results

### Modeling human cell adaptation to essential gene impairment *in vitro*

**Figure 1:**
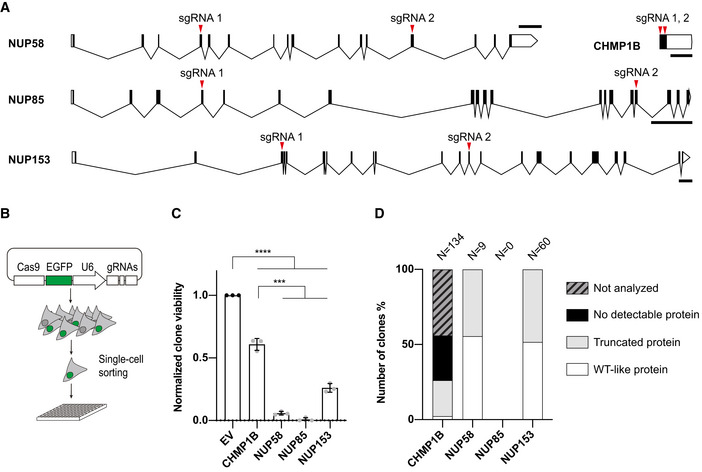
*NUP58*, *NUP85,* and *NUP153* are essential genes in human HAP1 cells Schematic representation of *NUP58*, *CHMP1B*, *NUP85,* and *NUP153* genes (transcript IDs reported: *NUP58* ENST00000381736.8, *CHMP1B* ENST00000526991.3, *NUP85* ENST00000245544.9, *NUP153* ENST00000537253.5). Exons shown as black boxes, introns as connecting black lines, and gRNA target sites are indicated with red triangles. Scale bars, 2 kb.Diagram detailing experimental method for isolation of mutant clones. After transfection with eGFP Cas9 plasmid carrying dual gRNA, GFP+ HAP1 cells were individually sorted into 96‐well plates. A total of 10 plates were prepared for each biological replicate (*n* = 3).Relative cellular viability following Cas9‐induced gene editing. Reported number of colonies was normalized based on viability of HAP1 cells transfected with an empty vector (EV) control (dots). Error bars indicate mean of SD from three biological replicates (number of colonies for EV rep1=242/rep2=238/rep3=201; CHMP1B rep1=134/rep2=151/rep3=128; NUP58 rep1=11/rep2=13/rep3=15; NUP85 rep1=0 /rep2=0/rep3=5; NUP153 rep1=61/rep2=55/rep3=60) (ordinary one‐way ANOVA ****P ≤ 0.00001; ns = non‐significant).Graph indicates the percentage of viable clones with either undetectable, truncated or wild‐type‐like proteins as assessed by Western blot analysis. Percentage of not analyzed colonies is reported after *CHMP1B* targeting. Results shown are from one of the three biological replicates in (C).

**Figure EV1:**
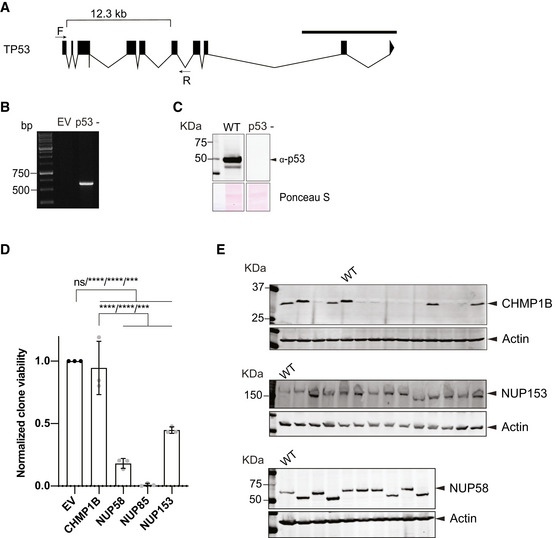
CRISPR‐Cas9 efficiency is independent of p53 activation Schematic representation of *TP53* gene (transcript ID: ENST00000615910.4). Highlighted is the 12.3 Kb region of the *TP53* locus that was targeted for Cas9 deletion. F (forward) and R (reverse) primers designed in exon 1 and introns 6–7, respectively, were used in B to verify deletion of the targeted region.PCR amplification of *TP53* locus in a p53‐ single cell‐derived clone using the above described primers after Cas9 gene editing. Distance if there is a deletion between the two gRNAs would result in a PCR product of ∼ 600 bp.Western blot analysis of p53‐ single cell‐derived clone shown in B (right). WT was loaded as a control (left). Ponceau staining was used as loading control. Both samples were treated with doxorubicin at 400 nM for 4 hours before harvesting.Relative cellular viability following Cas9‐induced gene editing in HAP1 p53‐ cell line. The experiment was performed as described in Fig 1C using three experimental replicates. Black bars indicate SD (number of colonies for EV rep1=252/rep2=303/rep3=214; CHMP1B rep1=202/rep2=361/rep3=181; NUP58 rep1=51/rep2=62/rep3=29; NUP85 rep1=2/rep2=0/rep3=5; NUP153 rep1=109/rep2=145/rep3=93)(ordinary one‐way ANOVA; ****P ≤ 0.00001, EV vs NU153 ***P = 0.0003, CHMP1B vs NUP153 ***P = 0.0007, ns = non‐significant).Representative CHMP1B, NUP153 and NUP58 immunoblots of single cell‐derived clones from the experiment described in Fig 1D. The NUP58 blot displayed includes all 9 samples analyzed in Fig 1D. Proteins are indicated by arrow heads. Actin was used as loading control.

To generate hypomorphic alleles of essential genes, we first sought to confirm the essentiality of candidates *NUP58*, *NUP153*, and *NUP85* in the human chronic myeloid leukemia near‐haploid cell line HAP1. To test essentiality, we disrupted each gene individually using CRISPR‐Cas9 with dual gRNA to introduce deleterious mutations at two unique target sites per gene. Target sites were prioritized according to their presence in all reported transcript isoforms and by domains in close proximity with conserved regions or known to be required for protein–protein interactions/NPC assembly (Fig 1A). To isolate individual clones, cells were transfected with a single vector co‐expressing Cas9, eGFP, and dual gRNAs, after which GFP‐positive cells were seeded individually into multi‐well plates (Fig 1B). To quantify cellular viability following disruption of NPC genes, single‐cell‐derived colonies were counted two weeks after seeding and compared with control clones (cells transfected with vectors harboring either no gRNAs [empty vector, EV] or gRNAs targeting the expressed non‐essential gene *CHMP1B* Hart *et al*, 2015). While a large proportion of cells remained viable following EV and *CHMP1B* gene editing, disruption of *NUP* alleles led to a drastic and statistically significant decrease in viable colony formation (Fig 1C). This reduced viability in *NUP*‐edited cells was not a consequence of p53‐mediated apoptosis resulting from Cas9‐induced DNA damage, since qualitatively similar results were obtained in a *TP53*‐null HAP1 cell line (Fig EV1A–D). Of the few colonies recovered, we performed Western blot on all viable NUP58 (9) and NUP153 (60) clones, as well as over half of the CHMP1B colonies (75) (data from one representative independent experimental replicate are shown in Fig 1D). NUP85 could not be tested since no viable colonies were isolated after disruption of this gene. While 53% of all analyzed CHMP1B clones were viable despite complete loss of the corresponding protein, we were unable to find any viable *NUP*‐targeted colonies displaying complete loss of NPC proteins. Indeed, viable NPC‐targeted colonies either retained WT‐migrating protein bands or expressed truncated NUP proteins (in approximately 44.4 and 43.8% of the NUP58 and NUP153 clones, respectively; Figs 1D and EV1E), suggesting that complete gene loss is lethal. Taken together, these data suggest that *NUP58*, *NUP85*, and *NUP153* are essential for viability of HAP1 cells.

### Mutant *NUP58* alleles are hypomorphic

**Figure 2:**
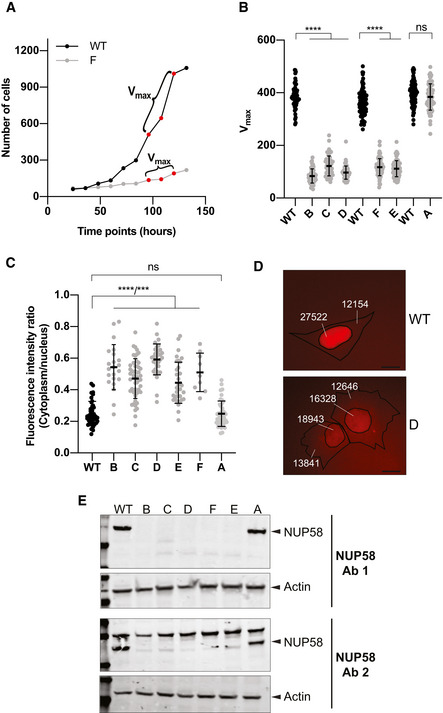
Cas9 targeting of essential *NUP58* exons generates hypomorphic alleles Determination of maximum growth rate (Vmax) using a label‐free cell count‐based proliferation assay. Representative growth curves of WT and NUP58 mutant clone F are plotted using a semi‐log scale displaying total numbers of cells over time. Growth rates were calculated across the entire duration of the experiment using a sliding window of three time points.Representation of the highest growth rates (Vmax) for each independent cell line. Error bars indicate mean with SD (Welch’s *t*‐test; B, C, D, F, and E ****P ≤ 0.00001, ns = non‐significant). Growth rates of mutant clones were compared with the untransfected (WT) cell line seeded in the same plate. Each dot represents a technical replicate (as plotted from left to right: WT *n* = 62, B *n* = 63, C *n* = 61, D *n* = 63, WT *n* = 84, F *n* = 80, E *n* = 82, WT *n* = 90, A *n* = 78) of one independent experimental replicate performed.Quantification of active nuclear import of asynchronous controls or mutant clones using an NLS‐3XmCherry as reporter. Ratio of the cytoplasm vs nucleoplasm fluorescence intensity is plotted. Only cells with comparable overall fluorescence intensity are reported (see extended M&M for details). Dots represent analyzed cells (WT *n* = 39, B *n* = 22, C *n* = 50, D *n* = 34, E *n* = 34, F *n* = 9, A *n* = 42) of one independent experimental replicate performed. Error bars indicate mean with SD (Welch’s *t*‐test; ns = non‐significant, B, C, D, E ****P ≤ 0.00001, F ***P = 0.0001).Representative images of analyzed WT (top) and clone D (bottom) NLS‐3xmCherry stable cells. Mean intensity of fluorescence for cytoplasm and nucleoplasm for each cell is labeled. Scale bar, 20 μm.Western blot for NUP58 protein expression in mutant clones (B‐E) and control cells (clone A and WT). Whole‐cell lysate hybridized with customized antibody 1 (Ab1, top panel) and antibody 2 (Ab2, bottom panel). Please see Appendix Fig S2A for detailed description of the used antibodies. Actin was used as loading control. Source data are available online for this figure.

Since the *NUP*‐targeted sites were located in close proximity to essential exons and conserved across all transcripts, we asked whether these truncated mutants would display impaired protein function and could therefore be used to dissect mechanisms of human cell adaptation to the drug‐mimicking impairment of essential genes. *NUP58* was selected for all subsequent experiments as its editing showed a greater impact on cell viability compared with *NUP153* disruption (Fig 1C). Since hypomorphic alleles confer partial loss of gene function, we expected mutant clones to exhibit decreased cellular fitness and impaired active nuclear import/export of large cargos (> 40 kDa) (Strambio‐De‐Castillia *et al*, 2010; Ma *et al*, 2012; Kabachinski & Schwartz, 2015). To test this, HAP1 cell line was first stably transduced with a NLS‐3XmCherry construct, encoding for a large cytoplasmic cargo of ∼ 84 kDa (see Appendix M&M for details) and then used to isolate freshly generated single cell‐derived NUP58 mutants as described in Fig 1. Clones were immediately analyzed for their fitness (measured by quantitative growth rate, Fig 2A and B), nuclear import ability (Fig 2C and D), and presence of hypomorphic NUP58 mutations (Fig 2E). All analyzed early‐derived NUP58 clones showing a decreased NUP58 expression had a concomitant impairment in nuclear import and fitness. WT clones (represented by clone A in Fig 2) showing a robust NUP58 expression did not display growth or nuclear import defects, suggesting that single‐cell clonal amplification does not affect cellular fitness or nuclear import capacity. Taken together, these observations suggested that the generated *NUP58* mutant alleles were hypomorphic and conferred reduced NUP58 activity, as assessed by decrease in cellular fitness and/or reduced ability to shuttle cargo from cytoplasm to nucleus. Moreover, they support the idea that CRISPR‐Cas9 can be used to generate mutant alleles of *NUP58* that are hypomorphic and suitable for modeling targeted drug therapy in human cells.

### Hypomorphic alleles generate low abundance, truncated proteins via exon skipping

**Figure 3:**
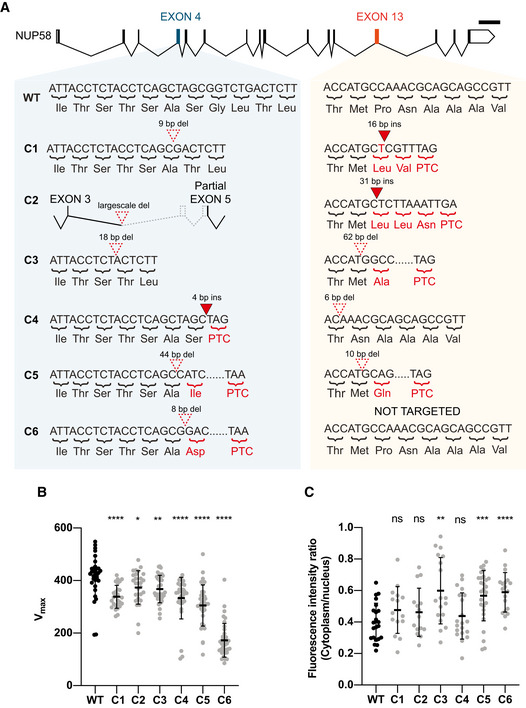
Generation of NUP58 mutant clones Diagrams detailing *NUP58* mutations in mutant HAP1 clones. Nucleotide and amino acid sequences of exons 4 and 13 surrounding the gRNA target site are shown for wild‐type (WT) and NUP58 mutant clones. Insertions are highlighted as closed red triangles and deletions as open dotted triangles over the nucleotide sequence. Amino acid substitutions and PTC resulting from nonsense mutations are indicated in red. A large‐scale deletion (large‐scale del) present in C2 and affecting part of intron 3–4, exon 4, intron 4–5, and part of exon 5 is represented with dashed lines.Representation of the highest growth rates (Vmax) for each technical replicate for each indicated cell line. Error bars indicate mean with SD (Welch’s *t*‐test; C1, C4, C5 and C6 ****P ≤ 0.00001, C2 *P = 0.0202, C3 **P = 0.0034). Each dot represents a technical replicate (WT *n* = 35, C1 *n* = 27, C2 *n* = 28, C3 *n* = 34, C4 *n* = 32, C5 *n* = 35, C6 *n* = 38) of one independent experimental replicate performed.Analysis of active nuclear import using an NLS‐3XmCherry as reporter as described in Fig 2C and D. Dots represent analyzed cells (WT *n* = 22, C1 *n* = 15, C2 *n* = 15, C3 *n* = 18, C4 *n* = 20, C5 *n* = 27) of one independent experimental replicate performed. Error bars indicate mean with SD (Welch’s *t*‐test; ns = non‐significant, C3 **P = 0.0015, C5 ***P = 0.0001, C6 ****P ≤ 0.00001).

To study adaptation of mammalian cells to the presence of *NUP58* hypomorphic alleles, we generated again fresh NUP58 mutant clones using WT HAP1 cells as described in Fig 1. Since overall viability was extremely low after CRISPR‐Cas9 targeting of *NUP58*, we seeded ∼ 3,000 single cells in order to identify multiple viable clones that were coding hypomorphic alleles. We expected mutant clones to exhibit decreased cellular fitness (Fig 2B), so we selected the six smallest viable clones for subsequent amplification (designated C1–C6). Whole‐exome sequencing at passage 1 (P1, please see Appendix M&M for detailed description of clone derivation) confirmed the presence of indel mutations within the *NUP58* genomic locus in all six clones, with mutations mapping to the Cas9 cleavage regions of all targeted sequences (Fig 3A). Premature termination codons (PTCs) were introduced in at least one of the two targeted exons in each clone, and large‐scale deletions surrounding the targeted nucleotides were also observed (C2 and C5). As expected, all clones displayed impaired growth compared with WT HAP1 cells (Fig 3B). To check for nuclear import defects, clones were stably transduced with NLS‐3XmCherry and analyzed as described in Fig 2C and D. As shown in Fig 3C, while some clones did not show significant impairment of trafficking, C5 and C6 exhibited a significant defect in ability to import NLS‐3XmCherry into the nucleus. Moreover, C3 showed a bimodal distribution of nuclear import capacity, with some cells able to shuttle NLS‐3XmCherry as effectively as the WT control, while others displayed substantial impairment. Since clones had to be transfected and selected for the introduction of the reporter, this result suggested that mutant cells might be capable of rapid evolution in response to *NUP58* hypomorphic alleles, perhaps accounting for the lack of significant difference in some clones. To test the generality of these findings, we also targeted *NUP58* in the colorectal HCT116 (near‐diploid) cell line. As shown in Appendix Fig S1A and B, we were able to isolate a HCT116 mutant clone encoding biallelic mutations that introduced a PTC in *NUP58* and resulted in decreased cellular fitness.

**Figure 4:**
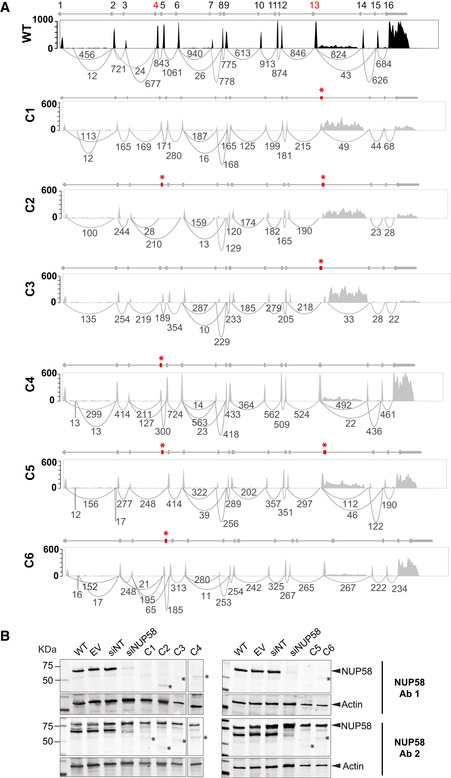
Alternative exon usage resulted in novel NUP58 protein variants Sashimi plots indicating combined exon usage of *NUP58* mRNA in HAP1 WT and mutant clones across four biological replicates. Numbers of reads per exon–exon junction are indicated below introns. WT canonical isoform is colored in black; mutant isoforms in gray. *NUP58* gene structure is reported over the sashimi plot; red asterisks and exons highlight the positions of the identified mutations.Western blot for NUP58 protein expression in mutant clones and control cells. Whole‐cell lysate hybridized with customized antibody 1 (Ab1, top panel) and antibody 2 (Ab2, bottom panel). Blots were loaded with wild‐type HAP1 cell line (WT), empty vector (EV), non‐targeting siRNA (siNT), and NUP58 targeting siRNA (siNUP58), followed by NUP58 mutant clones (C1/C2/C3/C4 in left panels, C5/C6 in right panels). Actin was used as loading control. Asterisks indicate possible NUP58 alternative isoforms. Source data are available online for this figure.

**Figure EV2:**
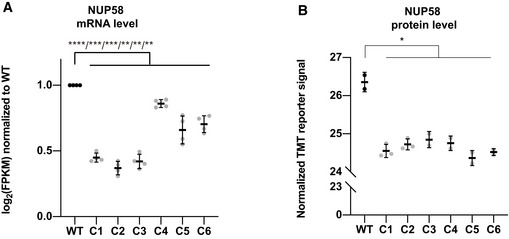
mRNA and protein levels quantification in HAP1 mutant clones Quantification of *NUP58* mRNA expression based on RNA sequencing dataset. Log₂ of FPKM (fragments per kilobase per million reads mapped) was normalized to wild type (WT). Mean with SD is plotted as a black bar representing the mean value of four independent experimental replicates performed (*n* = 4). (Welch’s *t*‐test; C1 ****P ≤ 0.00001, C2 ***P = 0.0001, C3 ***P = 0.0002, C4 **P = 0.0025, C5 **P = 0.0076, C6 **P = 0.0026).NUP58 protein levels quantified by TMT mass spectrometry. Normalized TMT reporter signals are displayed for mutant and control samples. Mean with SD is plotted as a black bar representing the mean value of three independent experimental replicates performed (*n* = 3). (Welch’s *t*‐test; C1 *P = 0.0233, C2 *P = 0.0352, C3 *P = 0.0253, C4 *P = 0.0235, C5 *P = 0.0155, C6 *P = 0.0408).

The hypomorphic alleles described above each contained PTCs or large deletions in the *NUP58* coding region. As shown in Figs EV1E and 2E, some of the NUP58 clones displayed faster migrating NUP58 bands relative to control lines, suggesting that the mutant alleles could generate truncated isoforms of NUP58 protein. Indeed, it was recently reported that mammalian cells can bypass Cas9‐induced nonsense mutations and large deletions *via* exon skipping, which results in the generation of partially functional truncated proteins (Mou *et al*, 2017; Chen *et al*, 2018; Smits *et al*, 2019). We therefore sought to identify potential NUP58 alternative isoforms by performing bulk RNA sequencing on the mutant clones at P1. As shown in Figs 4A and EV2A, all mutant clones exhibited decreased total *NUP58* read counts in respect to WT control, suggesting that the introduced mutations destabilized *NUP58* mRNA. Moreover, complete skipping of the mutated exons was evident in some clones (Fig 4A). All isoforms present in C2 and C5 skipped exon 4 and terminated the mRNA sequence at exon 13, possibly using the intron between exon 13 and 14 as the 3’ UTR. In contrast, all isoforms of C1 and C3 terminated the *NUP58* mRNA at exon 13, while C4 and C6 instead skipped the mutated exon 4 in only a subset of transcripts. It is important to note that since exons 4 and 5 are 150 and 138 nucleotides long, respectively, their deletion does not result in a frameshift downstream to the skipped exons. In accordance with RNA‐seq data, mutant alleles gave rise to truncated proteins that were present at lower abundance than the full‐length NUP58 in the WT background (Fig 4B and Appendix Fig S2A for antibodies binding sites). Tandem mass tag (TMT) isobaric labeling‐based quantitative proteomics analysis performed at P1 confirmed that the truncated proteins were present at lower steady‐state levels than in WT (Fig EV2B). Similar destabilization of the mutant mRNA and truncated protein was also observed in the HCT116 clone (Appendix Fig S2B–E). Taken together, these data showed that hypomorphic *NUP58* alleles carried PTCs and large deletions giving rise to truncated proteins with exon skipping and/or alternative stop codons and 3’UTRs. Truncated NUP58 proteins were expressed at lower levels than in WT, likely as a consequence of reduced mRNA and/or protein stability.

### Upregulation of karyopherins underlies early adaptation to decreased NUP58 activity

**Figure 5:**
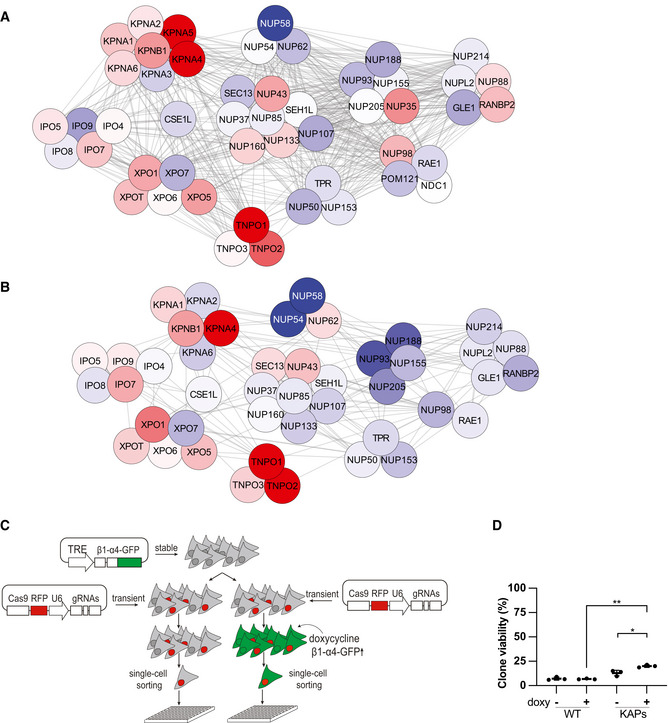
Upregulation of KPNB1 and KPNA4 underlies early adaptation to *NUP58* mutations A–BCombined median fold‐change in RNA (A) and protein (B) levels between NUP58 mutant clones and WT cells (calculated using *n* = 4 RNA‐seq and *n* = 3 mass spectrometry datasets). Data were visualized using Cytoscape. White: comparable average expression to WT; red: upregulated relative to WT; blue: down‐regulated relative to WT. Nodes belonging to the same subcomplex are grouped; edges represent physical interactions (see M&M for details). Transcripts and protein abundance changes in individual clones are reported in Fig EV3.CDiagram detailing experimental method for experiment performed in (D). A stable cell line carrying KPNB1‐KPNA4‐eGFP and a WT cell line were transfected with eRFP Cas9 plasmid carrying dual gRNA. Cell populations were either treated with doxycycline (doxy +) or left untreated (doxy −) and individually sorted into 384‐well plates. A total of 2 plates were prepared for each replicate.DCellular viability expressed as number of viable colonies following Cas9‐induced gene editing of *NUP58* in WT cell line and in a cell line stably overexpressing KPNB1‐KPNA4‐eGFP (KAPs). The experiment was performed using three independent replicates and as described in (C). Error bars indicate mean with SD (Welch’s *t*‐test; **P = 0.0017, *P = 0.0325).

**Figure EV3:**
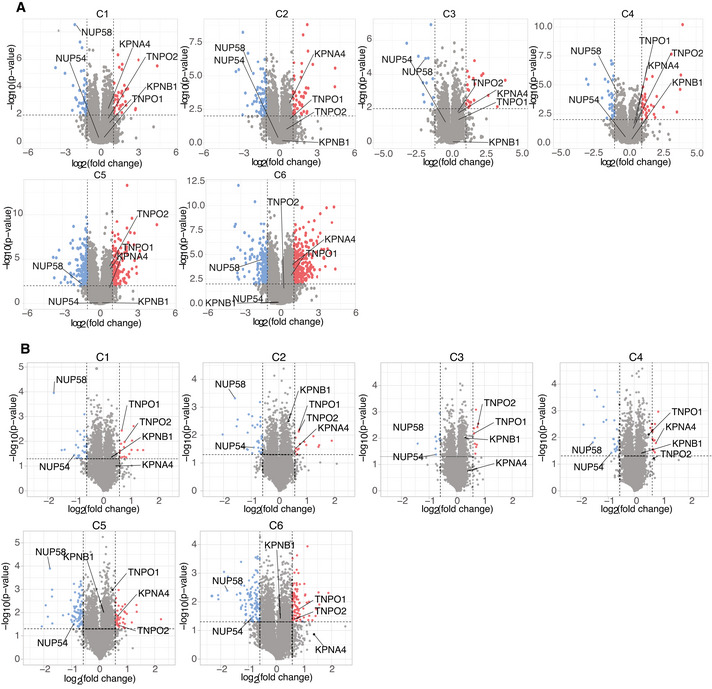
Differential expression of nuclear pore complex and nuclear transport genes in individual HAP1 mutant clones A–BVolcano plots reporting the level of mRNA (A) and protein (B) in each clone (C1 to C6) as a ratio relative to the WT control line. Red and blue dots indicate upregulated and down‐regulated genes/proteins, respectively. Selected relevant genes/proteins are labeled. For transcriptome = *P*‐value < 0.01, fold‐change > 2; for proteome = *P*‐value < 0.05, fold‐change > 1.5. We noted there is a discrepancy between the mRNA and the protein levels of NUP54. We think this is a consequence of the selective degradation of NUP54 at the protein level. Indeed, it is well known that proteins of NPC subunits are often degraded if their interacting partner(s) is absent or the stoichiometric ratio of the complex is otherwise out of balance (Boehmer *et al*, 2003). So in this case, while *NUP54* mRNA abundance is as in control cells, NUP54 protein is unstable and degraded as it cannot interact with its binding partner NUP58.

To search for genetic adaptive changes in response to *NUP58* hypomorphic alleles, we looked for genetic mutations common to all clones. As shown in Appendix Fig S3A, exome sequencing performed at P1 failed to detect possible adaptive mutations, suggesting that initial survival of NUP58 clones could be driven by transcriptome or proteome changes. To look for such changes, RNA‐seq and mass spectrometry datasets were analyzed for genes and proteins that were commonly and specifically up‐ or down‐regulated in the mutants. In addition to facilitating nuclear/cytoplasmic transport, the NPC plays a key role in controlling the expression of several developmental and transcriptional regulatory genes (Lupu *et al*, 2008; Raices & D'Angelo, 2012; Iwamoto *et al*, 2015; Sakuma & D'Angelo, 2017). Accordingly, a large fraction of such genes was differentially expressed in the NUP58 mutant clones (Appendix Fig S3B). Since expression changes in these genes likely reflect perturbations in NPC activity rather than adaptation to its loss of function, we instead focused our analysis on genes belonging to the nuclear transport family. To this end, we first calculated the median fold‐change relative to WT across four biological RNA‐seq experiments and then visualized common up‐ or down‐regulated genes using HumanNet network (PMID 30418591) (Fig 5A for median expression and Fig EV3A for gene changes at the individual clone level). Similar analyses were performed using three biological TMT replicates, focusing on common up‐ or down‐regulated proteins involved in molecular transport (visualized as a proteins physical interactome network [PPI] PMID 18823568–28514442) (Fig 5B displays median values, Fig EV3B presents protein changes at the individual clone level). Both analyses revealed a generalized downregulation of NPC components that were functionally related to or known to physically interact with NUP58 (Chug *et al*, 2015; Koh & Blobel, 2015). In parallel, TNPO1, TNPO2, KPNB1, and KPNA4 proteins belonging to the karyopherins family were upregulated in most clones. We observed a similar downregulation of NUP58‐interacting NPC proteins and upregulation of TNPO1, TNPO2, and KPNA4 in the HCT116 NUP58 mutant clone at P1 (Appendix Fig S4A and B). To independently confirm these results, we reanalyzed the TMT dataset and selected differentially expressed proteins which are known to genetically interact with the NPC in yeast (Costanzo *et al*, 2016). As shown in Appendix Fig S3C, proteins involved in nuclear–cytoplasm transport were differentially expressed, further supporting our previous conclusion that NUP58 clones upregulated karyopherins at P1. Karyopherins are transport receptors that mediate the import/export of macromolecules through the nuclear pore by physically interacting with FG nucleoporins (Zilman *et al*, 2007; Terry & Wente, 2009; Tan *et al*, 2018). To test whether transient upregulation of karyopherin is sufficient to increase the viability of freshly generated single‐cell NUP58 mutant clones, KPNB1‐KPNA4 genes under the control of a doxycycline‐inducible promoter (KPNB1‐KPNA4‐GFP plasmid) were integrated in a wild‐type HAP1 cell line (Fig 5C). As shown in Fig 5D, induction of KPNB1‐KPNA4 karyopherin overexpression significantly increases the viability of the NUP58 mutant clones. We also noted a slight viability increase in absence of doxycycline treatment. This is expected as the doxycycline expression system is documented to be leaky with reported basal expression of transgenes in absence of antibiotic (Meyer‐Ficca *et al*, 2004; Pham *et al*, 2008). Taken together, these data suggest that early adaptation to partial loss of NUP58 function is brought about by physiological changes in the form of upregulation of the transporter proteins that physically interact with the FG permeability barrier, thereby supporting cargo shuttling across the nucleoplasm and the cytoplasm.

### Long‐term adaptation to NUP58 disruption relies on stabilization of mutant isoforms

**Figure 6:**
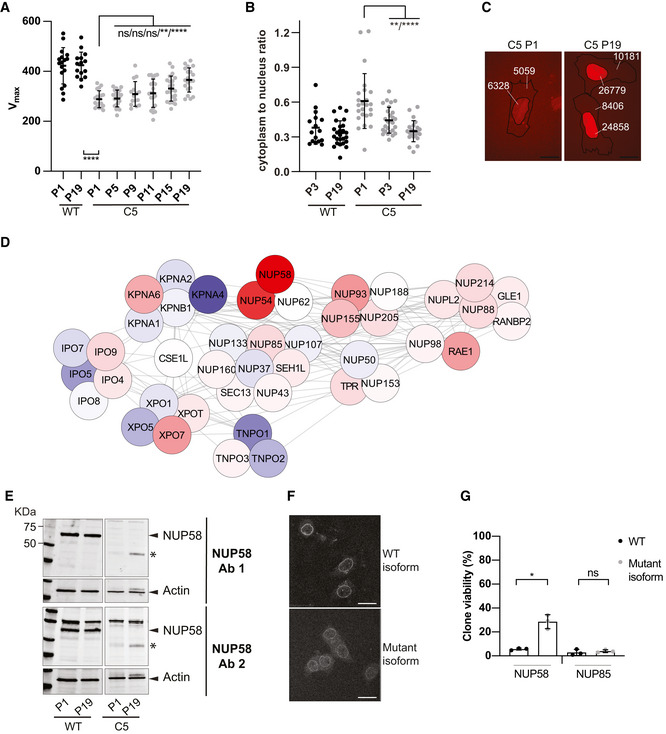
NUP58 mutant isoforms are stabilized upon fitness gain Maximum growth rates of NUP58 mutant clone C5 during long‐term culture. Each dot represents a technical replicate (*n* = 22). Error bars indicate mean with SD (Welch’s *t*‐test, ns: non‐significant, **P = 0.0030, ****P ≤ 0.00001).Improvement in active nuclear import upon cell passaging. Analysis was performed as in Fig 2C. Dots represent analyzed cells (WT P3 *n* = 16, WT P19 *n* = 25, C5 P1 *n* = 24, C5 P3 *n* = 27, C5 P19 *n* = 23) of one independent experimental replicate performed. Error bars indicate mean with SD (Welch’s *t*‐test; **P = 0.0037, ****P ≤ 0.00001).Representative images of analyzed C5 P1 (left) and C5 P19 (right) NLS‐3xmCherry stable cells. Mean intensity of fluorescence for cytoplasm and nucleoplasm for each cell is labeled. Scale bar, 20 μm.Cytoscape visualization of protein abundance changes between C5 P19 and C5 P1. Red nodes indicate proteins that were upregulated upon passaging; blue nodes indicate proteins that were down‐regulated upon passaging. White nodes display proteins with comparable expression levels at both early and late passage.NUP58 protein expression in C5 at P1 and P19. Blots were performed using antibody 1 (Ab1, top) or antibody 2 (Ab2, bottom). Actin was used as loading control. The band corresponding to the full‐length NUP58 is indicated by a black arrow with possible alternative isoform by an asterisk.Alternative NUP58 isoform localizes to the NPC. Representative images of HCT116 cell line showing localization of WT NUP58 protein (top WT isoform, z‐stack = 10) and of an isoform lacking exon 4 and ending at exon 13 (bottom mutant isoform, z‐stack = 11). Scale bar, 20 μm.Cellular viability following Cas9‐induced gene editing of native *NUP58* (left) or *NUP85* (right) in WT cell line (black circles) and in a cell line overexpressing NUP58 mutant isoform (gray circles). The experiment was performed using three independent experimental replicates. Error bars indicate mean with SD (Welch’s *t*‐test; *P = 0.0194, ns = non‐significant). Source data are available online for this figure.

**Figure EV4:**
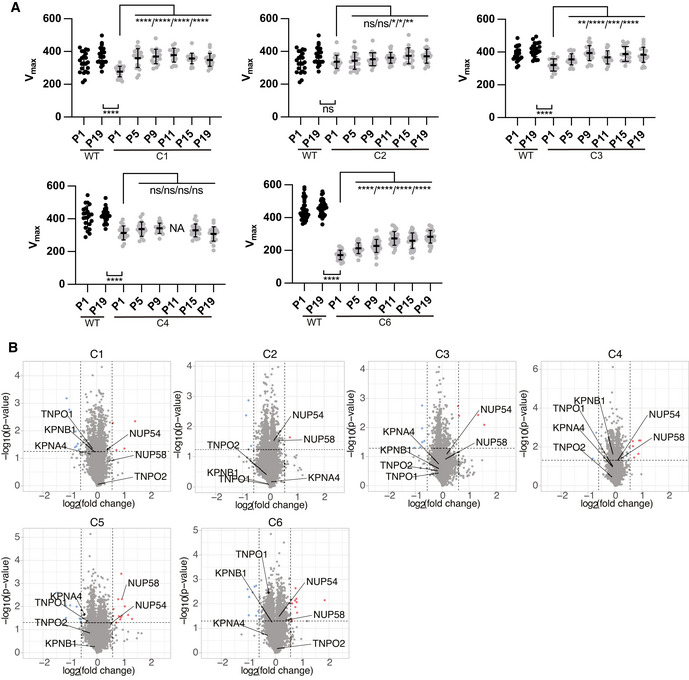
Long‐term fitness recovery and proteome changes in HAP1 mutant clones Growth rates of passaged mutant HAP1 cell lines and matched controls. WT cell lines were analyzed at P1 and P19 only. NUP58 mutant clones were analyzed at the indicated passage numbers. Each dot represents a technical replicate (*n* = 22) of one experimental replicate performed. Error bars indicate mean with SD (Welch’s *t*‐test; ns = non‐significant, **P ≤ 0.0030, ****P ≤ 0.00001).Volcano plots reporting the level of protein in each clone at P19 (C1 to C6) as a ratio relative to cell lines at P1. Red and blue dots indicate upregulated and down‐regulated genes/proteins, respectively. Select relevant genes/proteins are labeled. For transcriptome = *P*‐value < 0.01, fold‐change > 2; for proteome = *P*‐value < 0.05, fold‐change > 1.5.

**Figure 7:**
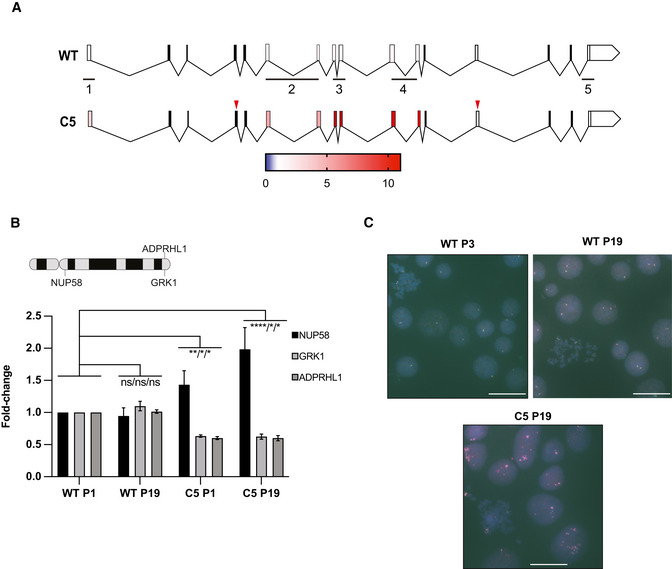
NUP58 is stabilized upon focal amplification of its coding sequence Comparison of *NUP58* mRNA abundance between late (P19) and early (P1) passage in wild‐type cells (WT, top) and clone C5 (bottom). mRNA was quantified using qPCR and five independent probe sets amplifying different regions of *NUP58* mRNA. Annealing of the probe sets is indicated at the bottom of the *NUP58* structure. Relative mRNA abundance between P19 and P1 is color‐coded for each exon analyzed (according to the color bar shown below). Red triangles highlight the position of the identified mutations in C5.Diagram at the top represents G‐banding pattern of chromosome 13 and highlights chromosome locations of tested genes (*NUP58*, *GRK1*, *ADPRHL1*). Graph at the bottom reports qPCR results for indicated genes performed on the gDNA of indicated samples at the indicated passage number. The experiment was performed using three independent experimental replicates. Error bars indicate mean with SD (2‐way ANOVA; *P = 0.02, **P = 0.0083, ****P ≤ 0.00001, ns = non‐significant).Representative images of *NUP58* gene‐specific FISH hybridization for control HAP1 cell lines (WT P3‐WT P19) and mutant C5 at P19. Each dot represents a *NUP58* locus of cluster thereof. Scale bar, 31.3 μm.

**Figure EV5:**
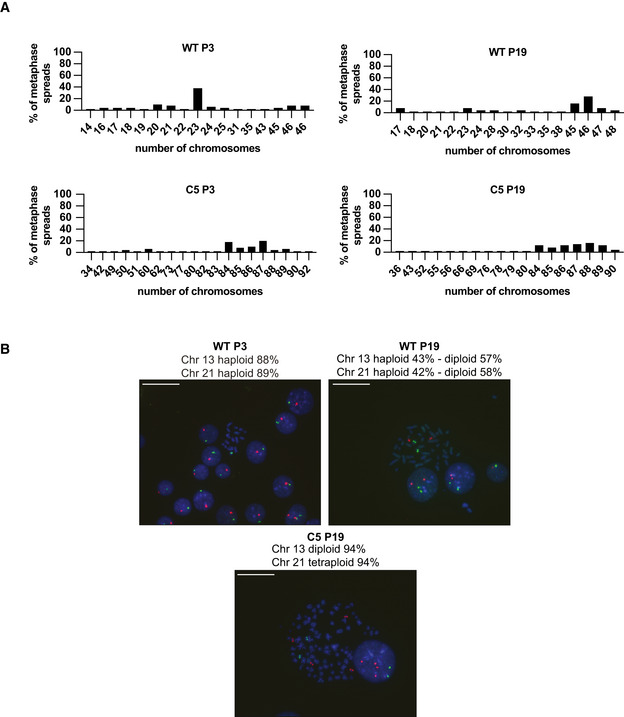
Karyotype analysis for mutant clone C5 Chromosome counts from metaphase spreads of HAP1 control lines (WT P3–WT P19) and mutant C5 at P19 (*n* = 50).Representative images of chromosome‐specific FISH hybridization for HAP1 control cell lines (WT P3–WT P19) and mutant C5 at P19. Green label Chr 13; orange label Chr 21. Scale bar, 31.3 μm.

Early adaptation by karyopherins overexpression may be a viable mechanism for human cells to survive the immediate deleterious effects of NUP58 disruption. However, cellular fitness remained severely impaired, suggesting that this initial adaptive change might be transient and swept by mutations conferring higher fitness. To identify subsequent adaptation mechanisms that more effectively recover cell function, NUP58 mutant clones (C1–C6) were serially cultured for ∼ 40 generations (20 passages in culture). Fitness was assessed *via* measurement of population growth rates at regular intervals. As shown in Figs 6A and EV4A, and Appendix Fig S5A most of the passaged mutant clones were able to increase their growth rates within the experimental time frame. Clones with smaller fitness defects at P1 show smaller fitness improvement during passaging, consistent with the declining adaptability concept (e.g., PMID: 25815007). HAP1 WT also showed a non‐significant fitness gain upon passaging, thereby suggesting that the observed fitness increase is not due to adaptation to the experimental conditions. Similarly, the HCT116 NUP58 mutant clone also displayed fitness improvement upon serial passages (Appendix Fig S5B). Taken together, these observations suggested that an adaptive mutation conferring an increased fitness recovery was acquired during the passaging and swept in the population, significantly improving growth rate beyond that achieved by early‐stage overexpression of karyopherins. Indeed, the nuclear import ability of C5 was improved upon passaging, further confirming that this second adaptive mutation had increased cellular benefits (Fig 6B and C). To identify candidate adaptive mechanisms, quantitative mass spectrometry analysis was performed on all clones at passage 19 (P19) and concomitant passage 1 (P1) samples. Among all clones, C5 was selected as representative clone for in‐depth analysis since it carries mutations in both exons 4 and 13, making it one of the most crippled alleles. Comparing fold‐change in C5 cellular transport genes between P1 and P19, we observed that P19 passaged clones increased NUP58 protein expression, stabilized the NPC, and down‐regulated expression of TNPO1, TNPO2, and KPNA4 (Fig 6D). Similar observations were also made when analyzing other HAP1 clones as well as the HCT116 clone (Fig EV4B and Appendix FigS5C). These results suggested that initial overexpression of karyopherins was not driven by genetic changes but rather physiological adaptation and rewiring of the transcriptome. Western blot analysis of C5 cells at P1 and P19 also confirmed that the truncated NUP58 protein was stabilized upon passaging (Fig 6E), suggesting that the mutant isoform is functional at low levels of expression that must be eventually increased to restore fitness. Similar results were also observed in some of the other clones (C1, C2, C3, C6 Appendix Fig S6A). In agreement with above data, an exogenous NUP58 isoform mimicking the mutations present in C5 was able to localize to the nuclear envelope, suggesting that this mutant allele does indeed remain functional (Fig 6F). To further support this, a HAP1 cell line stably expressing the NUP58 mutant isoform (2XGFP‐NUP58 mut plasmid) was subjected to CRISPR/Cas9 gene editing targeting the native form of *NUP58*. Specifically, exon 4 and exon 14, which are only present in the endogenous *NUP58,* were concomitantly targeted and the cell population was single‐cell plated. As shown in Fig 6G, cells overexpressing mutant NUP58 were able to significantly form more viable colonies than controls. However, overexpression of mutant NUP58 did not increase the viability of freshly generated NUP85 clones, suggesting that overexpression of mutant NUP58 specifically drives fitness gain of NUP58 mutant clones but not of other NPC mutations. Increased expression of the truncated NUP58 protein in P19 C5 correlated with significantly increased mRNA levels of several *NUP58* exons (Fig 7A). Gain in mRNA expression could result from mutations that increase mRNA stability or from copy number variation of the affected locus. To explore the latter possibility, we checked the copy number of chromosome 13, which encodes NUP58, and of *NUP58* locus. While chromosome 13 was not present in extra numerary copies in respect to the basal ploidy (Fig EV5A and B), *NUP58* locus but not its surrounding regions were amplified (Fig 7B and C). This result suggests that a focal amplification of the region encoding NUP58 was responsible for its upregulation. Taken together, these data suggest that fitness improvement was driven by copy number variation of *NUP58* locus and increased expression of NUP58 mutant isoform.

## Discussion

In this manuscript, we tested the ability of human cells to adapt to partial inactivation of essential genes and dissected early and late adaptation mechanisms in order to shed light into the interplay between genetic adaptations and non‐genetic mechanisms (such as phenotypic plasticity and non‐stochastic switching). Since essential genes are priority molecules for drug treatment, but target inactivation is typically only partial due to pharmacological and physiological constraints (Masui *et al*, 2013; Maeda & Khatami, 2018), we also put forward our approach as a genetic method to mimic drug treatment and elucidate mechanisms of emerging resistance to targeted therapy. Previous reports have indicated that early PTC or indels generated by genome editing can be bypassed by exon skipping, resulting in mutant alleles that are generally expressed or translated at lower levels (Smits *et al*, 2019; Tuladhar *et al*, 2019). We therefore used CRISPR‐Cas9 to edit conserved domains of essential NPC genes before selecting slow‐growing colonies to enrich for mutant lines expressing partially functional alleles. With this approach, we generated six mutant cell lines that were able to express mutant *NUP58* alleles by skipping exons containing PTCs or indels. The skipped exons were multiple than 3‐nt long, suggesting that targeting such exons could be a strategy to generate hypomorphic alleles of essential genes. Accordingly, no NUP85 viable mutants were retrieved where the CRISPR/Cas9 system targeted exons that were not multiple of 3 nts. Generated *NUP58* alleles were hypomorphic since mutant cells exhibited substantially decreased cellular fitness associated with reduced nuclear import ability. Contrary to initial expectations, impaired protein function was not driven by the absence of key domains arising from exon skipping, but rather an overall decrease in protein expression level following gene mutation. Indeed, upregulation of mutant protein was sufficient to increase the viability of NUP58 mutant colonies, suggesting that the mutant isoforms are functional. Therefore, caution should be applied when assessing the function of protein domains by observing only cellular phenotype resulting from domain‐specific mutations.

To dissect the role of phenotypic plasticity and genome changes in early and late evolutionary adaptation, we analyzed the transcriptome and the proteome of all early and late passaged mutant lines. We discovered that early adaptation correlated with upregulation of karyopherins, a class of NUP58 functionally related proteins which bind its FG repeats for nuclear trafficking. Accordingly, several negative and positive genetic interactions are documented in budding yeast between karyopherins and NPC components (Wilmes *et al*, 2008; Costanzo *et al*, 2016; Kuzmin *et al*, 2018). Moreover, it was shown that the C‐terminal FG domain of the NUP58‐NUP54 complex changes conformation based on the local concentration of karyopherins (Koh & Blobel, 2015). Specifically, in presence of karyopherins, NUP58‐NUP54 FG repeats switch from a constricted to a dilated conformation to allow the passage of the nuclear transporter proteins through the pore channel. Therefore, in the presence of high local karyopherins concentration, the pore dilation is such that karyopherins can diffuse through the pore in a more FG‐independent manner (Koh & Blobel, 2015). While this strategy can sustain cell viability, transport might become leaky as less dependent on the FG‐NUP domains. Accordingly, karyopherins upregulation was observed as transient solution characterized by impaired cellular fitness and reduced nuclear import. These findings are in accordance with our observation and could mechanistically explain how a slight upregulation of karyopherins could facilitate the active transport of cargoes through the pore in mutants of NUP58 lacking the FG repeats.

There is renewed debate in the literature about the extent to which genetic adaptations and non‐genetic mechanisms (such as phenotypic plasticity and non‐stochastic switching) can coexist, about their epistatic relationship and how they could influence subsequent evolutionary changes. (Ancel, 2000; Price *et al*, 2003; Paenke *et al*, 2007; Wund, 2012). For instance, certain genotypes might be compatible with cellular changes brought about by certain non‐genetic adaptations, while other genotypes might not. These evolutionary concepts have great implications for biomedical research and in particular to emergence of cancer drug resistance, cancer immune evasion, and metastasis (Bell & Gilan, 2020). And while these factors are key contributing elements to treatment failure and patient demise, it remains debated whether they can be attributed entirely to genetic mutations or whether non‐genetic mechanisms can also drive them (Salgia & Kulkarni, 2018). Indeed, a large body of literature links genetic changes with drug resistance. However, recent observations point toward pervasive non‐genetic changes as another trick cancer cells have to achieve drug resistance (Bell & Gilan, 2020). For instance, epigenetic changes have been described to drive stable drug‐resistant phenotypes (Pisco *et al*, 2013; Milanovic *et al*, 2018). In other cases, the drug‐resistant phenotype was shown to be unstable and lost upon drug withdrawal (Knoechel *et al*, 2014; Sun *et al*, 2014). Moreover, in some triple‐negative breast cancer patients genetic adaptations and phenotypic plasticity were shown to coexist (Kim *et al*, 2018). It is therefore of paramount importance to understand the contribution of genetic adaptation and non‐genetic mechanisms in drug resistance and dissect their interplay by using *in vitro* model systems. In the current study, we set up to mimic drug treatment by generating an hypomorphic allele of an essential gene and dissecting its drug‐resistant mechanisms by letting the cells to adapt to the genetic insult. We showed that early non‐genetic mechanisms and phenotypic plasticity in the form of karyopherins upregulation can initially support proliferation of NUP58 mutant cells. However, these changes are not sufficient to restore long‐term fitness, which is instead increased by genetic changes in the form of focal amplification of mutant *NUP58* alleles. These observations suggested that initial upregulation of karyopherins is most likely driven by phenotypic plasticity within the transcriptome rather than acquisition of genome mutations or stable epigenetic marks in karyopherin regulatory elements. In this context, we propose that early phenotypic plasticity allows cells to survive the initial “drug” insult, thereby permitting subsequent genome changes and adaptive evolution to occur. Our study sheds new light into how a complex interplay between genetic adaptations and non‐genetic mechanisms can facilitate evolutionary processes. It also suggests that inhibition of early non‐genetic changes may limit subsequent emergence of drug resistance. Moreover, it shows that mimicking targeted therapy with genetic models *in vitro* may uncover additional opportunities to optimize treatment.

## Materials and Methods

### Cell culture, gene editing, and stable cell line generation

HAP1 (Horizon Discovery C859), HCT116 (ATCC CCL‐257), and HEK293T (ATCC) cells were cultured under standard conditions. gRNAs for target genes were designed using the CHOPCHOP tool (http://chopchop.cbu.uib.no; Appendix Table S1) and cloned into pCAGGS expression vector (generously provided by Dr. Norris Ray Dunn, Lee Kong Chian School of Medicine, Singapore) which contained either GFP, puromycin, or RFP resistance cassette. HAP1 and HCT116 were transfected using Lipofectamine 3000 Transfection Reagent (Invitrogen) and single cells were isolated using a BD FACSAria II 5 sorter or via infinity dilution, respectively (see Appendix M&M). Gene editing in individual single‐cell‐derived clones was confirmed by Sanger sequencing and Western blot. To generate stable cell lines, HAP1 cells were transduced with NLS‐3XmCherry, composed of 3 mCherry proteins fused head to tail and carrying a nuclear localization signal [NLS] at the N terminus (BioBasic Asia Pacific) or 2XGFP‐NUP58 mut and selected by cell sorting; cells transduced with KPNB1‐KPNA4‐GFP were selected in medium containing 10 µg/ml blasticidin (Invivogen) (Appendix M&M for plasmid construction).

### Western blot, siRNAs, and quantitative RT‐PCR

Cell lysates were prepared using RIPA lysis and extraction buffer (Thermo Fisher Scientific) and proteins were visualized using LI‐COR Odyssey Imaging System. Commercial and customized antibodies are described in Appendix Table S2 and Appendix Fig S2. RNA silencing was performed by transfecting cells with siRNAs SMARTpool (Dharmacon D‐001810‐10‐05 non‐targeting, L‐013864‐01‐0005 siNUP58) and Lipofectamine RNAiMAX Transfection Reagent (Thermo Fisher Scientific). RT‐qPCR was performed on a QuantStudio Real‐Time PCR System (Applied Biosystems) with Perfecta SYBR FastMix ROX 5000 (Research Biolabs). Data were analyzed using the ΔΔCt method (see Appendix for probes sequences).

### Live‐cell imaging

HAP1 and HCT116 cells were stably or transiently transfected with NLS‐3XmCherry (a large cytoplasmic cargo of ∼ 84 kDa, composed of 3 mCherry proteins fused head to tail and carrying a nuclear localization signal [NLS] at the N terminus, NLS‐3XmCherry) or pEGFP2‐Nup58 constructs (BioBasic Asia Pacific), respectively. The cells were then plated on μ‐Dishes (Ibidi; 35 mm, high glass bottom) that had been pre‐coated with 15 µg/ml of fibronectin (Sigma) before imaging on spinning disk confocal microscope 3D FRAP (Nikon). Images were subsequently analyzed using ImageJ. Wild‐type and mutant cells with similar total mean fluorescence intensity were compared.

### Growth rate

HAP1 or HCT116 cells were seeded into 384‐well plates at 400 or 250 cells per well, respectively. Cell counts were performed at regular intervals using a Cytation5 (Biotek) multimode plate reader and analyzed using Gen5 v3.3 software. Vmax was calculated as the largest V by interpolating 3 consecutive data points. For HCT116, the Vmax was normalized based on cell count. RNA and exome‐sequencing gDNA and total RNA were isolated from 4 million cells using FavorPrep™ Blood/Cultured Cell Genomic DNA Extraction Mini Kit (Favorgene) or RNeasy Mini Kit (Qiagen), respectively. Capture‐seq (whole exome) and cDNA libraries were prepared according to standard operating procedures and samples were run on HiSeq4000 2 × 151 bp Multiplex (Illumina).

### Metaphase preparation and ploidy classification

Cells grown to ∼ 80% confluency were treated with 100 ng/ml Colcemid solution (Gibco) for 3 h, collected by trypsinization, and centrifuged at 1,000 rpm for 10 min (Giam *et al*, 2020). Cell pellets were resuspended in 75 mM potassium chloride solution and incubated for 15 min in a 37°C waterbath. Next, a 1/10 volume of 3:1 methanol/acetic acid was added to the cells prior to centrifugation at 1,000 rpm for 15 min. Cells were then fixed by resuspension in 3:1 methanol/acetic acid solution, incubated for 30 min at room temperature, centrifuged at 1,200 rpm for 5 min, and finally washed once more with fixative. Cells were resuspended in a small volume of fixative, dropped onto clean glass slides, and allowed to air‐dry. For chromosome counting, metaphase spreads were stained with Giemsa stain (Gibco) and acquired using the fully automated Metafer imaging platform (MetaSystems). Chromosome numbers were scored manually using ImageJ.

### Fluorescent In Situ Hybridization (FISH)

Metaphase preparations (described above and in Giam *et al*, 2020) or interphase cells were dropped onto glass slides and co‐denatured with XA 13/18/21 AneuScore probe mix (MetaSystems) for chromosome copy number, or with NUP58‐20‐GR/NUP58‐20‐OR (Empire Genomics) for focal amplification detection. Slides were incubated at 75°C for 2 min before being placed in a humidified slide incubator (Eppendorf) at 37°C for 24 h. Following hybridization, slides were washed first in 0.4X SSC (pH 7.0) at 72°C for 2 min, then in 2× SSC/0.05% Tween 20 (pH 7.0) at room temperature for 30 s, rinsed briefly in distilled water, and mounted on microscope slides with fluorescence mounting media (Dako) together with DAPI. Cells were visualized using the automated Metafer imaging platform (MetaSystems).

### TMT labeling and mass spectrometry

Sample preparation, TMT labeling, and offline fractionation were performed as previously described (Backlund *et al*, 2020) with the following modifications. For TMT10, a gradient of 120 min was used. For TMT6, trapping was carried out with a constant flow of trapping solution (0.05% trifluoroacetic acid in water) and peptides were eluted via the analytical column running solvent A (0.1% formic acid in water, 3% DMSO) with a constant flow of 0.3 µl/min, with increasing percentage of solvent B (0.1% formic acid in acetonitrile, 3% DMSO) from 2 to 8% in 6 min, then 8 to 28% for a further 66 min, in another 4 min. from 28 to 38%, followed by an increase of B from 38–80% for 3 min and a re‐equilibration back to 2% B for 5 min. IsobarQuant (https://doi.org/10.1038/nprot.2015.101) and Mascot (v2.2.07) were selected for data processing. A Uniprot Homo sapiens proteome database (UP000005640) containing common contaminants and reversed sequences was used. The search parameters were as follows: carbamidomethyl (C) and TMT10 (K) (fixed modification), acetyl (N‐term), oxidation (M), and TMT10 (N‐term) (variable modifications). A mass error tolerance of 10 ppm was set for the full scan (MS1) and for MS/MS (MS2) spectra of 0.02 Da. Trypsin was selected as the protease with a maximum of two missed cleavages permitted. A minimum peptide length of seven amino acids and at least two unique peptides per protein were required for a protein identification. False discovery rate at both peptide and protein level was set to 0.01.

### Transcriptome and proteome data representation

Pre‐alignment quality control of sequence reads was performed using the FastQC tool. Adaptor trimmed sequence reads were aligned to the genome (Ensembl GRCh38.p13) using STAR mapper with default settings (PMID 23104886). A fragments per kilobase million (FPKM) quantification table was obtained using RSEM tool (PMID 21816040), and no particular data normalization was deemed necessary based on visualization of data distribution (boxplots and heatmap). For both mRNA and protein abundance, comparisons between unpassaged clones and wild‐type samples used two‐sample independent *t*‐tests with *P*‐values adjusted for multiple testing based on *q*‐values. For comparison between passaged and unpassaged clones, paired *t*‐tests were used to account for correlations within the same clones. Sashimi plots of exon usage in individual clones were drawn using the Gviz library in R Bioconductor after the bam files from replicate experiments were merged for each clone. Biological networks for the transcriptome and proteome data were visualized using Cytoscape (PMID: 14597658). The gene network for the transcriptomics data was obtained from the co‐expression‐based network in HumanNet v2 database (PMID 30418591), and the physical interaction network for the proteomics data was curated from multiple sources (including iRefIndex [PMID 18823568] and BioPlex 2.0 [PMID 28514442]).

### Data quantification

Statistical analysis was performed with GraphPad Prism v9.1.1 (GraphPad software Inc. USA). To determine statistical significance among investigated groups Welch’s *t*‐test, one‐way or 2‐way analysis of variance (ANOVA) with Tukey’s multiple comparison test or Dunnet’s multiple comparison test was used.

## Author contributions

GR conceived the study, supervised, and secured funding. AT, KL, and GR prepared the figures and wrote the manuscript. AT, CKW, YLC, and RF performed the experiments. AT, HC, and JL performed data analysis and visualization. All authors read and agreed to the content.

## Conflict of interest

The authors declare that they have no conflict of interest.

## Supporting information



Appendix

Expanded View Figures PDF

Source Data for Appendix

Source Data for Figure 2

Source Data for Figure 4

Source Data for Figure 6

## Data Availability

The mass spectrometry proteomics data have been deposited to the ProteomeXchange Consortium via the PRIDE partner repository with the dataset identifier PXD022255. The RNA‐seq data presented have been deposited in the Gene Expression Omnibus repository (https://www.ncbi.nlm.nih.gov/geo/) with the accession number GSE161061 (Reviewer token access: kzsrimuwfdgjxml). The exome‐sequencing data have been deposited in the NCBI Sequence Read Archive (SRA) (https://dataview.ncbi.nlm.nih.gov/object/PRJNA680690?reviewer=qfr48p1q56uvm8n6c8d0r4n2i5).
