# Supplementary material for: Non‐genetic and genetic rewiring underlie adaptation to hypomorphic alleles of an essential gene
Source: EMBO J. 2021 Sep 15;40(21):e107839. doi: 10.15252/embj.2021107839 (PMC8561638; doi:10.15252/embj.2021107839)
Supplement: Supplementary file 1 — Appendix [file EMBJ-40-e107839-s001.pdf]

Appendix

Non-genetic and genetic rewiring underlie adaptation to hypomorphic alleles of an essential gene

Altea Targa[1,2,3], Katherine E Larrimore[1,2], Cheng Kit Wong[1], Yu Lin Chong[1,2], Ronald Fung[1], Joseph Lee[5], Hyungwon Choi[5] and Giulia Rancati[1,2,3 #]

1. Institute of Medical Biology (IMB), Agency for Science, Technology and Research (A*STAR), Singapore 138648, Singapore
2. Skin Research Institute of Singapore (SRIS), Agency for Science, Technology and Research (A*STAR), Singapore 138648, Singapore
3. School of Biological Sciences, Nanyang Technological University, Singapore 637551, Singapore
4. Department of Medicine, Yong Loo Lin School of Medicine, NUS and National University Health System, Singapore, 119228, Singapore

Table of contents

**Appendix Materials and Methods**	**Page 1**
**Appendix Table S1**	**Page 1**
**Appendix Table S2**	**Page 3**
**Appendix Figures S1-S6**	**Page 4**

Appendix Materials and Methods

Appendix Table S1: gRNA sequences used in this study

Guide RNAs sequence	Forward	Reverse
**NUP58 exon 4**	TACATGCTCTGATGACTGAT	ATCAGTCATCAGAGCATGTA
**NUP58 exon 13**	AACGGCTGCTGCGTTTGGCA	TGCCAAACGCAGCAGCCGTT

**NUP85 exon 4**	TACATGCTCTGATGACTGAT	ATCAGTCATCAGAGCATGTA
**NUP85 exon 16**	CAGAAGGAGAGAAGCTGCGT	ACGCAGCTTCTCTCCTTCTG
**NUP153 exon 3**	GCATTCCCAATTGGCAGTTC	GAACTGCCAATTGGGAATGC
**NUP153 exon 13**	GGCAGTGAAGAACTGGTGAT	ATCACCAGTTCTTCACTGCC
**CHMP1B exon 1_gRNA 1**	CATCCGCCAGAAGAACCAGG	CCTGGTTCTTCTGGCGGATG
**CHMP1B exon 1_gRNA 2**	GAGTGCGCGAGTCGATGCAG	CTGCATCGACTCGCGCACTC
**TP53 exon 1**	AGGGAAGCGTGTCACCGTCG	CGACGGTGACACGCTTCCCT
**TP53 exon 5**	CTGAGCAGCGCTCATGGTGG	CCACCATGAGCGCTGCTCAG

Cell lines transfection and single cell plating

HAP1 and HCT116 were transfected using Lipofectamine3000 Transfection Reagent (Invitrogen) according to the manufacturer's protocol. 2 days post-transfection HAP1 GFP (or RFP) positive cells were single-cell sorted in 96-well plates using BD FACSAria II 5 Lasers cell sorter. Cells harvested were prepared for sorting by resuspending them in a suspension buffer (PBS / EDTA 5 mM / FBS) and filtered in a tube with cell strainer cap to detach cells aggregates. HCT116 cells were selected with 1 µg/ml of puromycin (Sigma-Aldrich) for 2 days, starting 24 hours post-transfection, and then single-cell diluted in 96-well plates. For transient overexpression of KPNB1-KPNA4-GFP in HAP1 cells, 24 hours post-transfection, karyopherin expression was induced by treatment with 0.5 µg/mL of Doxycycline (Sigma-Aldrich) for 48 hours. GFP/RFP positive cells were single-cell sorted in 384-well plates.

Plasmids

2XGFP-NUP58 mut plasmid was generated by inserting the PCR amplified 2XGFP-NUP58 mut (BioBasic custom made) into pLVX-azurite (Addgene #36086) using XbaI and BsmbI restriction sites. KPNB1-KPNA4-eGFP was cloned into pCW-Cas9-Blast (83481) using NEBuilder HiFi DNA Assembly. KPNB1 derived from pEGFP-importinB1 (P30478) and KPNA4-GFP from pcDNA3.1 KPNA4-eGFP (GenScript). P2A was added between the two expressing sequences.

Evolutionary experiment

Passage 1 (P1) was defined as the earliest time point after *NUP58* gene-editing and single-cell seeding, when the number of cells reached ~15 X 10^6 to be collected for RNAseq, exome sequencing. Assuming no cell death, P1 would correspond to ~23 generations from the initial mutant single cell generated. Starting from P1 the cells were splitted 1:4 at ~80% of confluence.

Appendix Table S2: List of antibodies used in this study:

**Antibodies**	**Concentration**
**Ab1 NUP58 (customized by GL Biochem)**	1:1000
**Ab2 NUP58 (customized by GenScript)**	1:500
**CHMP1B (sc514013)**	1:1000
**NUP85 (customized by GL Biochem)**	1:500
**NUP153 (ab24700)**	1:1000
**p53 (MA512571)**	1:200
**Actin (MA5-15739)**	1:1000
**IRDye 680RD (LI-COR Biosciences) anti-mouse or anti-rabbit**	1:5000

Customized NUP58 antibodies recognition sites:

- **GenScript NUP58 antibody**

MDENLPPVICQDVENLQKFVKEQKQVQEEISRMSSKAMLKVQEDIKALKQLLSLAANG
IQRNTLNIDKLKIETAQELKNAEIALRTQKTPPGLQHEYAAPADYFRILVQQFEVQLQQY
RQQIEELENHLATQANNSHITPQDLSMAMQKIYQTFVALAAQLQSIHENVKVLKEQYLGY
RKMFLGDAVDVFETRRAEAKKWQNTPRVTTGPTPFSTMPNAAAVAMAATLTQQQQPATG
HHHHHH

- **GL Biochem NUP58 antibody**

SSDKKSDKTGTRPED-Cys

- **GL Biochem NUP85 antibody**

TLVSDRFLRDYCERG

Appendix Figures S1-S6

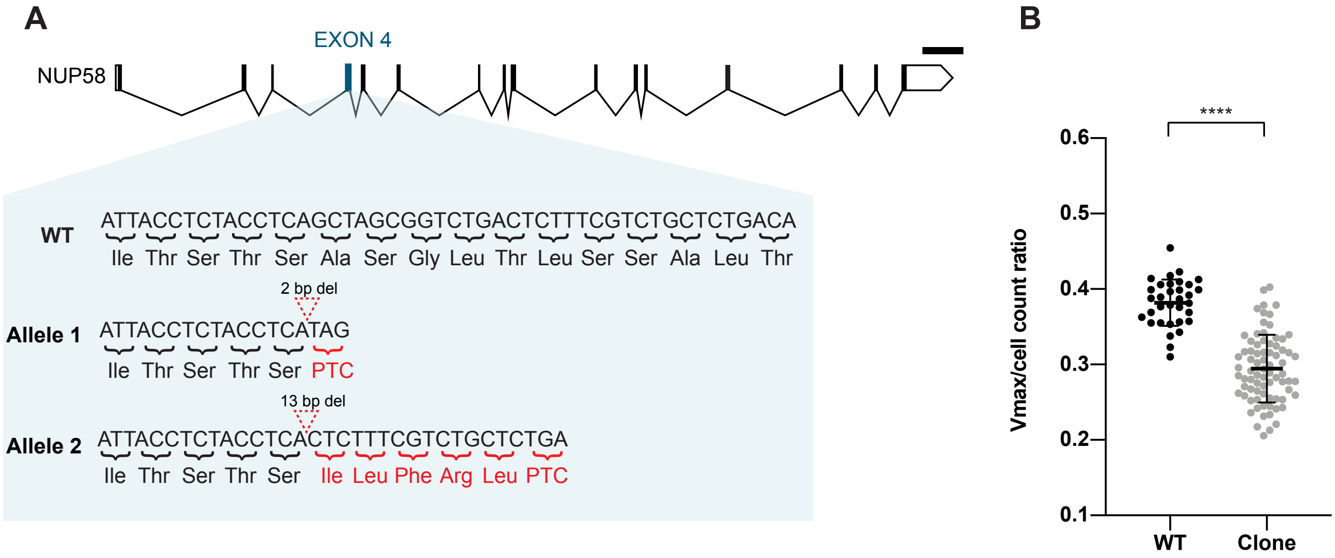

Appendix Figure S1.

A. Schematic representation of NUP58 mutations in the HCT116 clone. Nucleotide and amino acid sequences of exon 4 surrounding the gRNA target site are shown for wild-type (WT) and both alleles of the NUP58 mutant clone. Deletions are highlighted as open dotted triangles above the nucleotide sequence. Amino acid substitutions and PTC resulting from nonsense mutations are indicated in red.

B. Quantification of HCT116 WT and mutant clone maximum growth rate (Vmax) using a label-free cell count-based proliferation assay. Each dot represents a technical replicate. Error bars indicate mean with SD (Welch's t test; ****P ≤ 0.00001).

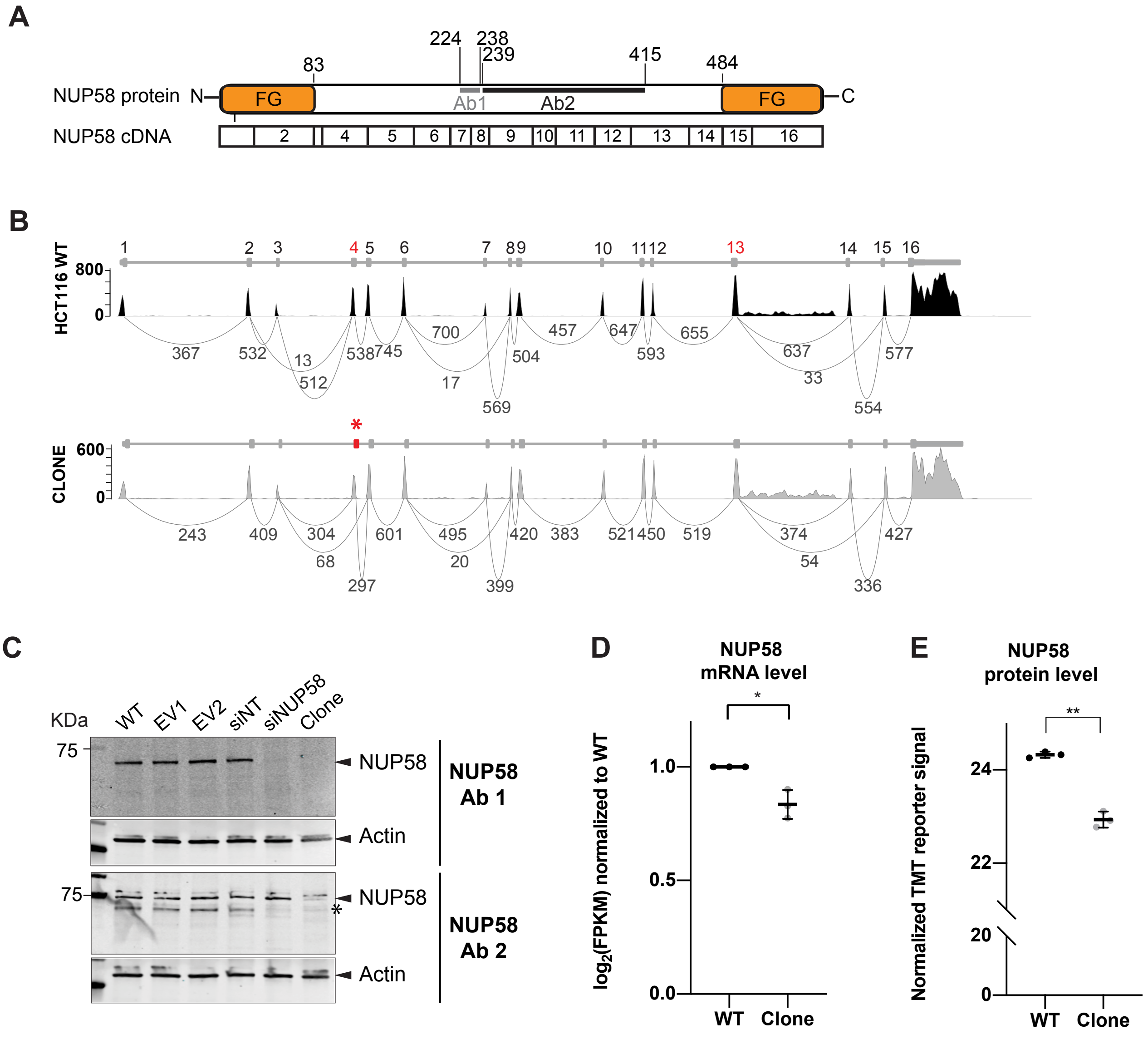

Appendix Figure S2.

A. Schematic diagram illustrating NUP58 recognition sites for antibodies Ab1 and Ab2. Top: NUP58 protein structure; sequences recognized by Ab1 and Ab2 are highlighted as grey and black bars, respectively. The N-terminal and C-terminal FG domains of NUP58 are highlighted in orange. Numbers over the NUP58 diagram indicate amino acid positions. Bottom: cDNA of NUP58 sequence matching the protein structure. Each box represents an exon.

B. Sashimi plots indicating combined exon usage of *NUP58* mRNA in HCT116 WT and mutant clone across four biological replicates. Numbers of reads per exon-exon junction are indicated below introns. WT canonical isoform is colored in black; mutant isoform in grey. *NUP58* gene structure is reported over the sashimi plot; red asterisks and exons highlight positions of the identified mutation.

C. Western blot for NUP58 protein expression in HCT116 mutant clone and control cells. Whole-cell lysate hybridized with customized antibody 1 (Ab1, top panel) and antibody 2 (Ab2, bottom panel). Blots were loaded with wild-type HCT116 cell line (WT), empty vector (EV), non-targeting siRNA (siNT), and NUP58 targeting siRNA (siNUP58), followed by NUP58 mutant clone. Actin was used as loading control. Asterisk indicate possible NUP58 alternative isoform.

D. Quantification of mRNA expression in HCT116 mutant clone based on RNA-sequencing dataset. Log_2 of FPKM (fragments per kilobase per million reads mapped) were normalized to wild-type (WT). Each dot represents one technical replicate. Error bars indicate mean with SD (Welch's t test; Clone *P = 0.0452).

E. NUP58 protein levels in HCT116 mutant clone quantified by TMT mass spectrometry. Normalized TMT reporter signals are displayed for mutant and control samples. Each dot represents a biological replicate. Black bars indicate error of mean with SD (Welch's t test; Clone **P = 0.0019).

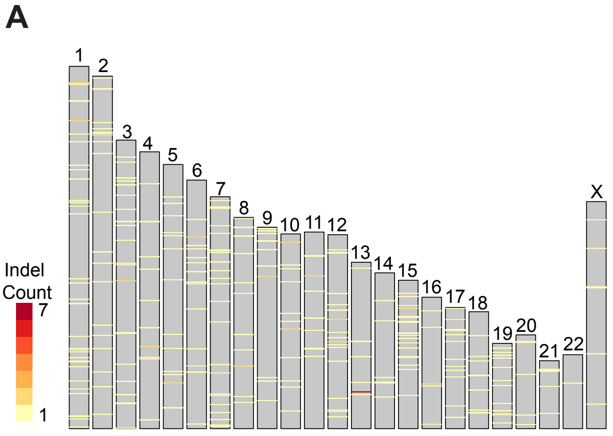

>3 mut/gene	Chr	INDEL
COL24A1	1	CCTTCTTCTCCCTTAAAAAAAAAAAAAAGTC
ICA1L	2	GAAGGAAGAAGTACATGCATGATTTTTTTTTTTTTTGC
AASDH	4	TTTTCGTCCCAAAAAAAAAAATCTCTCCA
RNF145	5	ATGttgttttttttttcttttttttttcttGGAGAAGACC
PTK7	6	AACGGTCGgtgtgtgtgtgtgtgttgtgggggag
HOOK3	8	ATGTGCTTTTTTTTTTTTTTTTAGAAAGAA
FAM135B	8	CAAGTTATGCCCTACAAAAAAAAAAAGAAAGAAAA
RABGAP1	9	TTTCATGTTCAAAAAAAAAAAAAGGACTCAATT
WDFY4	10	tgaaagagaccttttttttttttagatctcagcccc
ROM1	11	CGGCTGGTGGGGGGGGGGCTCCTGGTCGTC
CASP5	11	TCTTAACTGTTTTTTTTTTTTGTGGTTGTCTT
BLM	15	tagccAAGACTTTTTTTTTTTTTCCCTCAAAGAAA
BRD7	16	CCAGATCCTCTCTCTCTCTCTGCCAGCAGCCTCC
SLFN12L	17	AACACAGGCCCTTTTTGCAGGGAATTCTGGT
FHOD3	18	ACTGGCAGCAGAGAGAGAGAGGCGGCGGC
POP4	19	TTCTCCTGCCttttttttttttttttCAGTTATCCCC

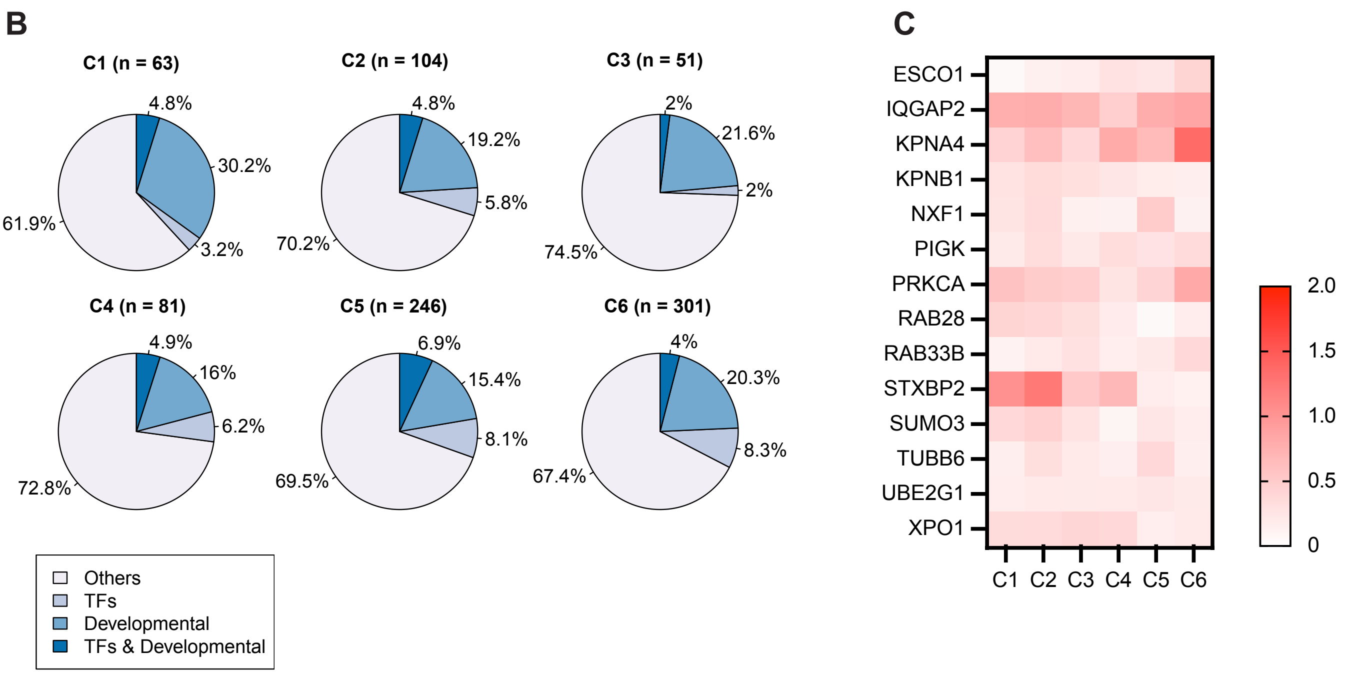

Appendix Figure S3

A. Chromosomes distribution of INDEL counts. The diagram on the left shows the localization of indels on each chromosome; the table on the right reports genes found mutated in >3 clones and details the nucleotides surrounding the mutation (highlighted in red, INDEL). The only two exome mutations present in all clones are encoded in *NUP58* and in *AASDH*, a beta-alanine activating enzyme. The latter mutation is unlikely to be adaptive in response to NUP58 inactivation for two reasons. First of all, there is no evidence in the literature that alanine metabolism is implicated in nuclear trafficking. Secondly, the *AASDH* mutation map in an A-rich and difficult to sequence region, suggesting that it might be a false positive. We also checked mutations that are present in at least 3/5 clones and found 15 of them. All of them map in difficult to sequence regions and are therefore likely false positive.

B. Piecharts displaying percentages of TFs and developmental proteins upregulated in the indicated NUP58 mutant clones at P1 in respect to control. ($p<0.05$, fold-change >1.25)
C. Heatmap displaying differentially expressed proteins involved in nuclear-cytoplasm transport and trafficking with log2FC >0.2 in at least 3/6 clones. To performed this analysis, we mapped all nuclear transport genes from human to yeast and identified their interactors from Cell Map data (Costanzo et al., 2016). We filtered the interactors based on p-value <0.01 and genetic interaction score >0.2. This yielded ~300 interactors that were mapped back from yeast to human. Please note that not all genes could be mapped due to lack of homologs between the two model systems. Among these differentially expressed genes, the karyopherins were the mainly represented class of proteins.

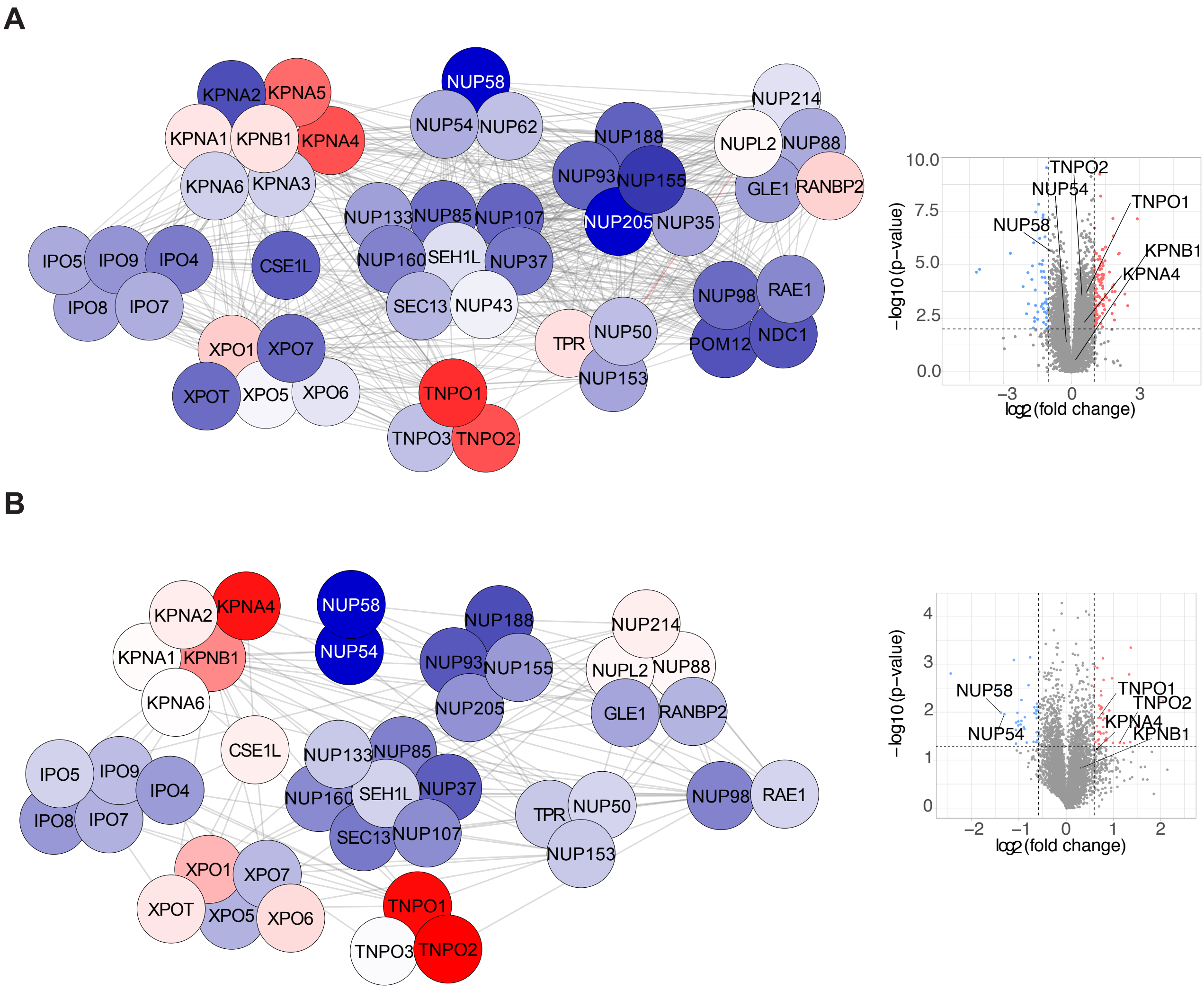

Appendix Figure S4

A - B. Fold-change in RNA (A) and protein (B) levels between NUP58 HCT116 clone and WT cells (calculated using n=4 RNA-seq and n=3 mass spectrometry datasets). On left data were visualized using Cytoscape. White: comparable average expression to WT; red: upregulated relative to WT; blue: downregulated relative to WT. Nodes belonging to the same subcomplex are grouped; edges represent physical interactions (see M&M for details). On right volcano plots reporting the level of mRNA (A) and protein (B) as a ratio relative to the wild-type control line. Red and blue dots indicate upregulated and downregulated genes/proteins, respectively. Select relevant genes/proteins are labelled. For transcriptome = p-value < 0.01, fold change > 2; for proteome = p-value < 0.05, fold change > 1.5.

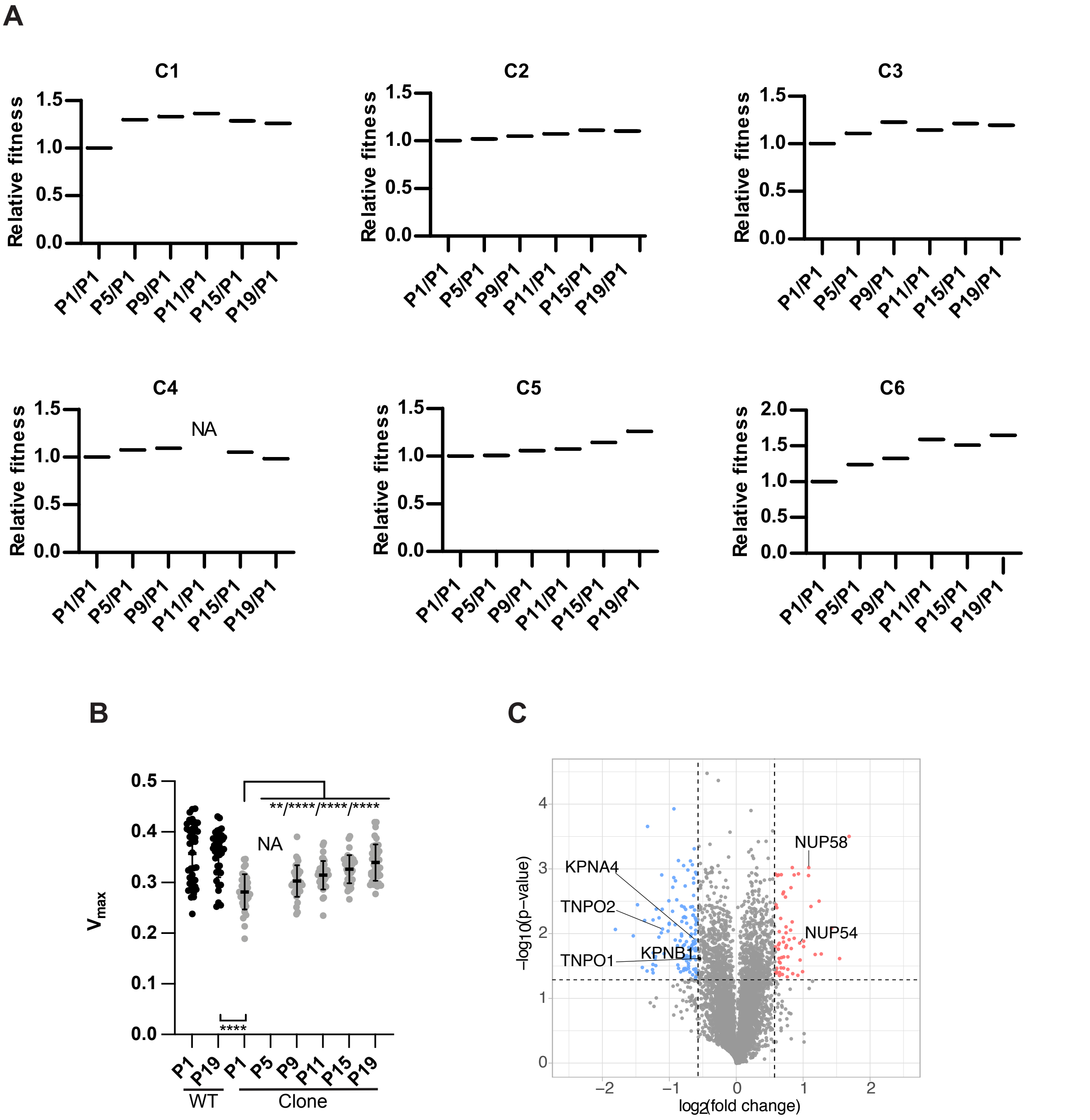

Appendix Figure S5

A. Relative fitness of indicated clones normalized to P1. Each line was calculated by averaging the growth rates (N >= 20) for each passage and then normalized it to the average growth rate at P1.

B. Maximum growth rate of NUP58 HCT116 mutant clone during long-term culture. Each dot represents a technical replicate. Error bars indicate mean with SD (Welch's t test, ns: non-significant, **P ≤ 0.0030, ****P ≤ 0.00001).

C. Volcano plots reporting the level of protein in HCT116 clone at P19 as a ratio relative to cell line at P1. Red and blue dots indicate upregulated and downregulated genes/proteins, respectively. Select relevant genes/proteins are labelled. For transcriptome = p-value < 0.01, fold change > 2; for proteome = p-value < 0.05, fold change > 1.5.

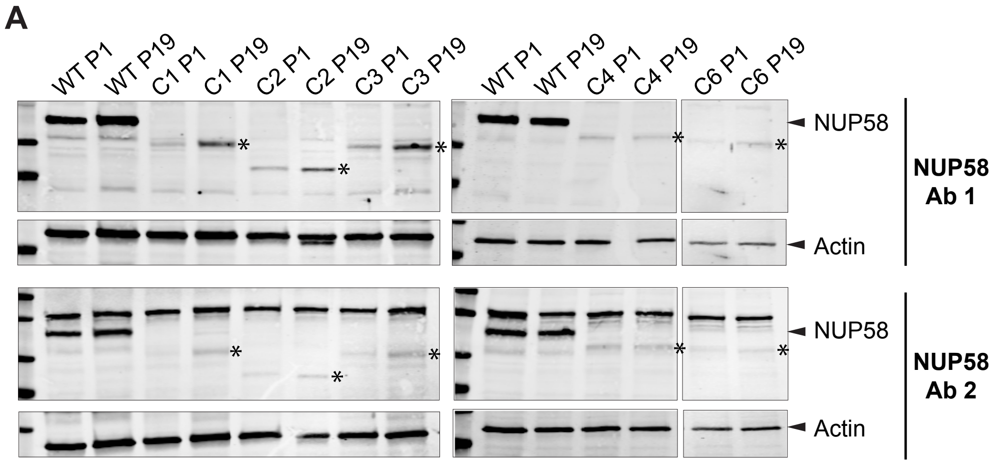

Appendix Figure S6

A. Western blot for NUP58 protein expression in mutant clones and control cells at P2 and P19. Whole-cell lysate hybridized with customized antibody 1 (Ab1, top panel) and antibody 2 (Ab2, bottom panel). Blots were loaded with wild-type HAP1 cell line (WT) followed by NUP58 mutant clones. Actin was used as loading control. The band corresponding to the full-length NUP58 is indicated by a black arrow with possible alternative isoform by an asterisk.
