# Supplementary material for: Non‐genetic and genetic rewiring underlie adaptation to hypomorphic alleles of an essential gene
Source: EMBO J. 2021 Sep 15;40(21):e107839. doi: 10.15252/embj.2021107839 (PMC8561638; doi:10.15252/embj.2021107839)
Supplement: Supplementary file 2 — Expanded View Figures PDF [file EMBJ-40-e107839-s004.pdf]

Expanded View Figures

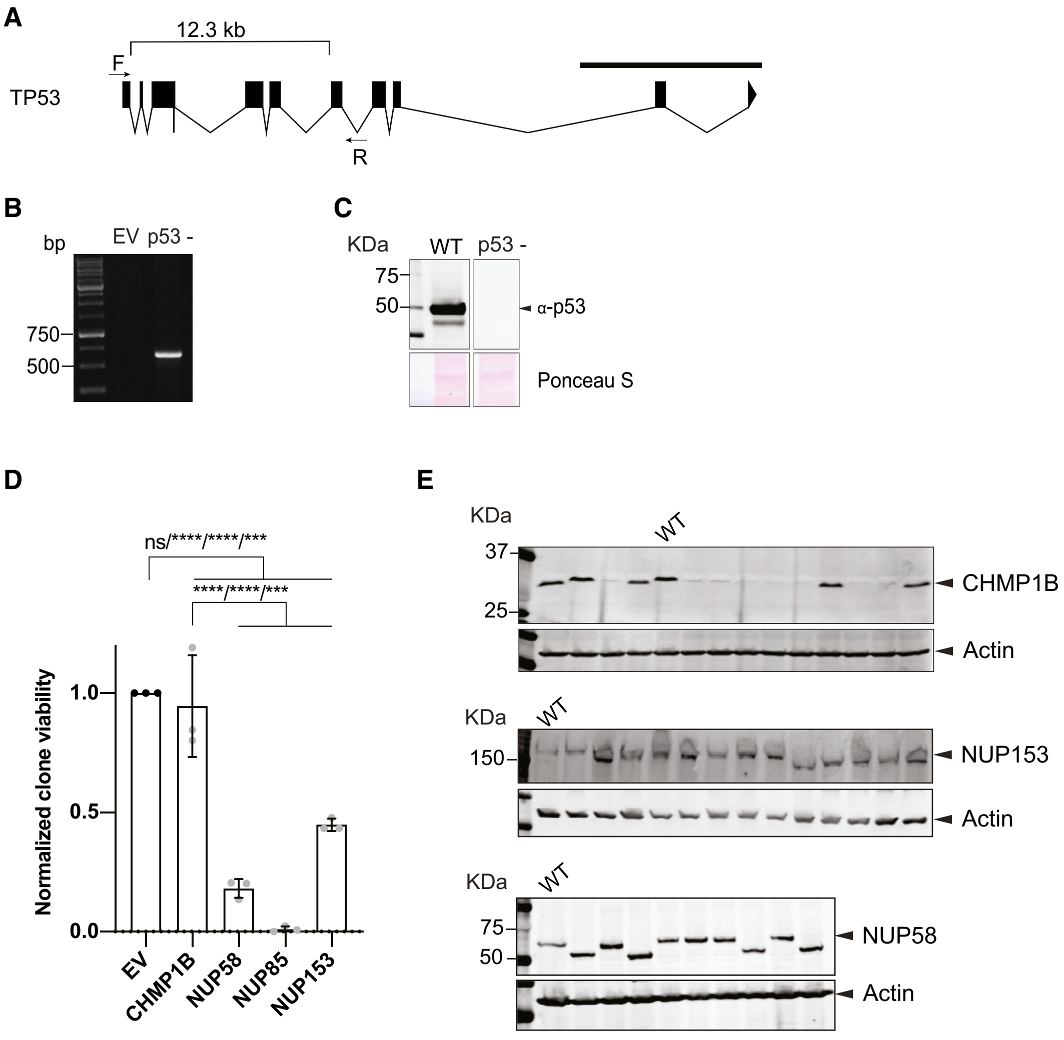

Figure EV1. CRISPR-Cas9 efficiency is independent of p53 activation.

A Schematic representation of *TP53* gene (transcript ID: ENST00000615910.4). Highlighted is the 12.3 Kb region of the *TP53* locus that was targeted for Cas9 deletion. F (forward) and R (reverse) primers designed in exon 1 and introns 6–7, respectively, were used in B to verify deletion of the targeted region.

B PCR amplification of *TP53* locus in a p53- single cell-derived clone using the above described primers after Cas9 gene editing. Distance if there is a deletion between the two gRNAs would result in a PCR product of ∼ 600 bp.

C Western blot analysis of p53- single cell-derived clone shown in B (right). WT was loaded as a control (left). Ponceau staining was used as loading control. Both samples were treated with doxorubicin at 400 nM for 4 hours before harvesting.

D Relative cellular viability following Cas9-induced gene editing in HAP1 p53- cell line. The experiment was performed as described in Fig 1C using three experimental replicates. Black bars indicate SD (number of colonies for EV rep1=252/rep2=303/rep3=214; CHMP1B rep1=202/rep2=361/rep3=181; NUP58 rep1=51/rep2=62/rep3=29; NUP85 rep1=2/rep2=0/rep3=5; NUP153 rep1=109/rep2=145/rep3=93)(ordinary one-way ANOVA; $****P \leq 0.00001$, EV vs NU153 $***P = 0.0003$, CHMP1B vs NUP153 $***P = 0.0007$, ns = non-significant).

E Representative CHMP1B, NUP153 and NUP58 immunoblots of single cell-derived clones from the experiment described in Fig 1D. The NUP58 blot displayed includes all 9 samples analyzed in Fig 1D. Proteins are indicated by arrow heads. Actin was used as loading control.

 © 2021 The Authors

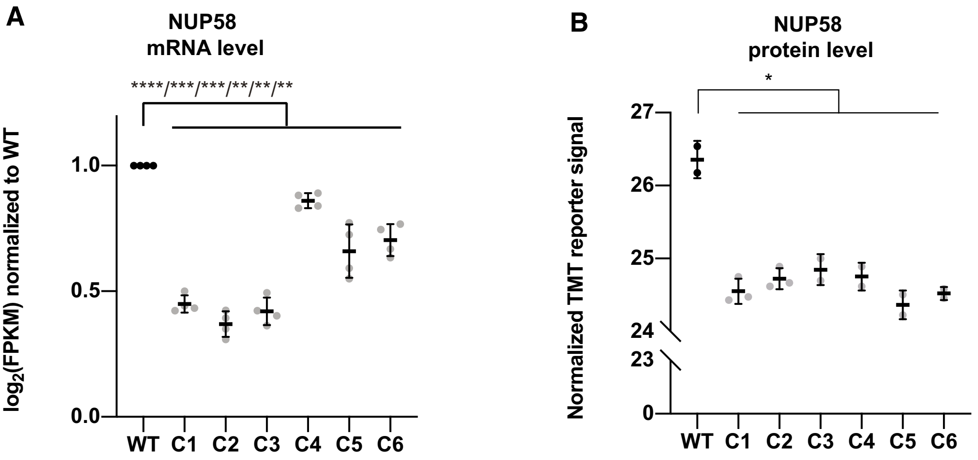

Figure EV2. mRNA and protein levels quantification in HAP1 mutant clones.

A Quantification of *NUP58* mRNA expression based on RNA sequencing dataset. Log_2 of FPKM (fragments per kilobase per million reads mapped) was normalized to wild type (WT). Mean with SD is plotted as a black bar representing the mean value of four independent experimental replicates performed (n = 4). (Welch's t-test; C1 ****$P \leq 0.00001$, C2 ***P = 0.0001, C3 ***P = 0.0002, C4 **P = 0.0025, C5 **P = 0.0076, C6 **P = 0.0026).

B NUP58 protein levels quantified by TMT mass spectrometry. Normalized TMT reporter signals are displayed for mutant and control samples. Mean with SD is plotted as a black bar representing the mean value of three independent experimental replicates performed (n = 3). (Welch's t-test; C1 *P = 0.0233, C2 *P = 0.0352, C3 *P = 0.0253, C4 *P = 0.0235, C5 *P = 0.0155, C6 *P = 0.0408).

© 2021 The Authors

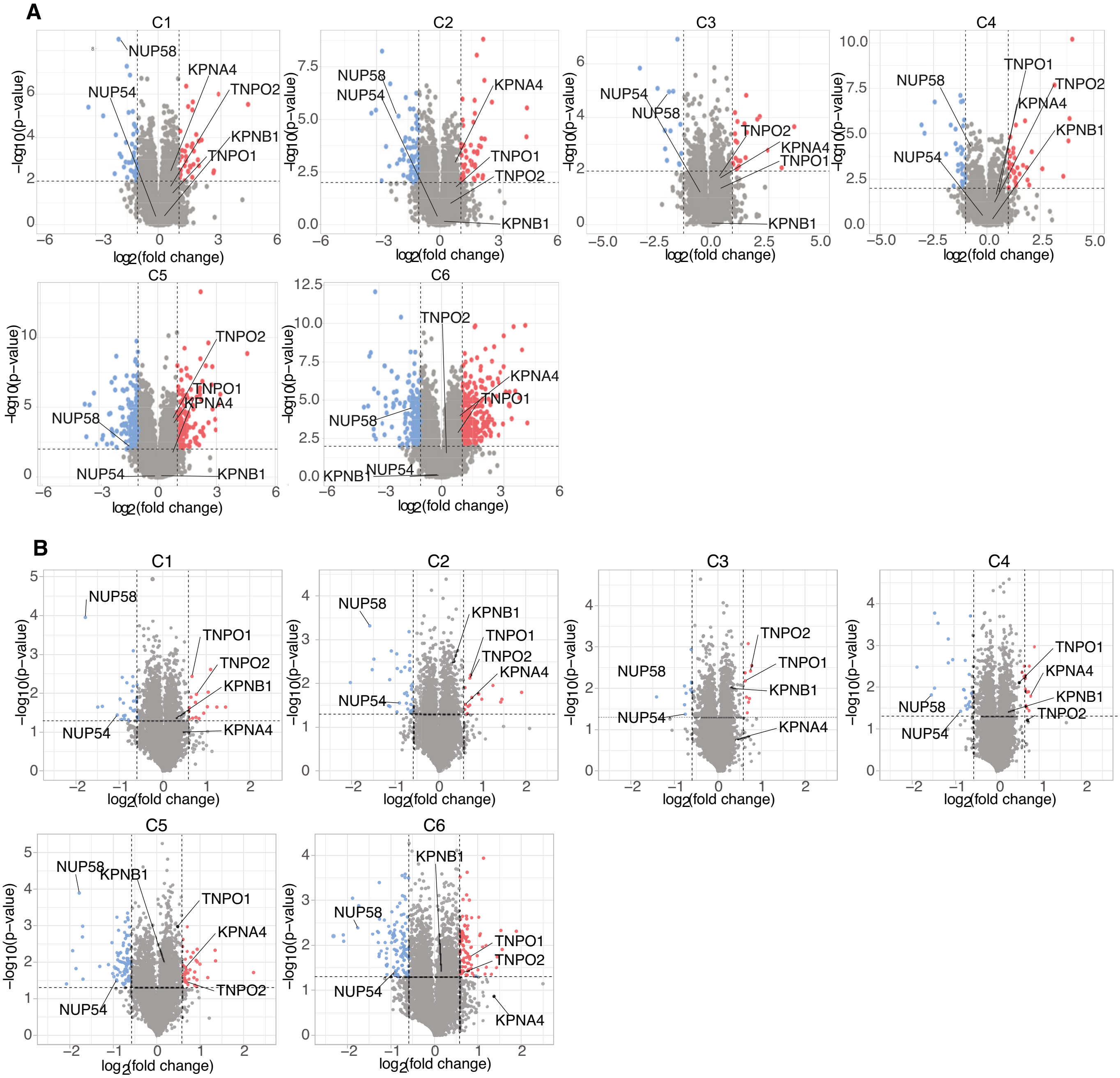

Figure EV3. Differential expression of nuclear pore complex and nuclear transport genes in individual HAP1 mutant clones.

A–B Volcano plots reporting the level of mRNA (A) and protein (B) in each clone (C1 to C6) as a ratio relative to the WT control line. Red and blue dots indicate upregulated and down-regulated genes/proteins, respectively. Selected relevant genes/proteins are labeled. For transcriptome = P-value < 0.01, fold-change > 2; for proteome = P-value < 0.05, fold-change > 1.5. We noted there is a discrepancy between the mRNA and the protein levels of NUP54. We think this is a consequence of the selective degradation of NUP54 at the protein level. Indeed, it is well known that proteins of NPC subunits are often degraded if their interacting partner(s) is absent or the stoichiometric ratio of the complex is otherwise out of balance (Boehmer *et al*, 2003). So in this case, while *NUP54* mRNA abundance is as in control cells, NUP54 protein is unstable and degraded as it cannot interact with its binding partner NUP58.

© 2021 The Authors

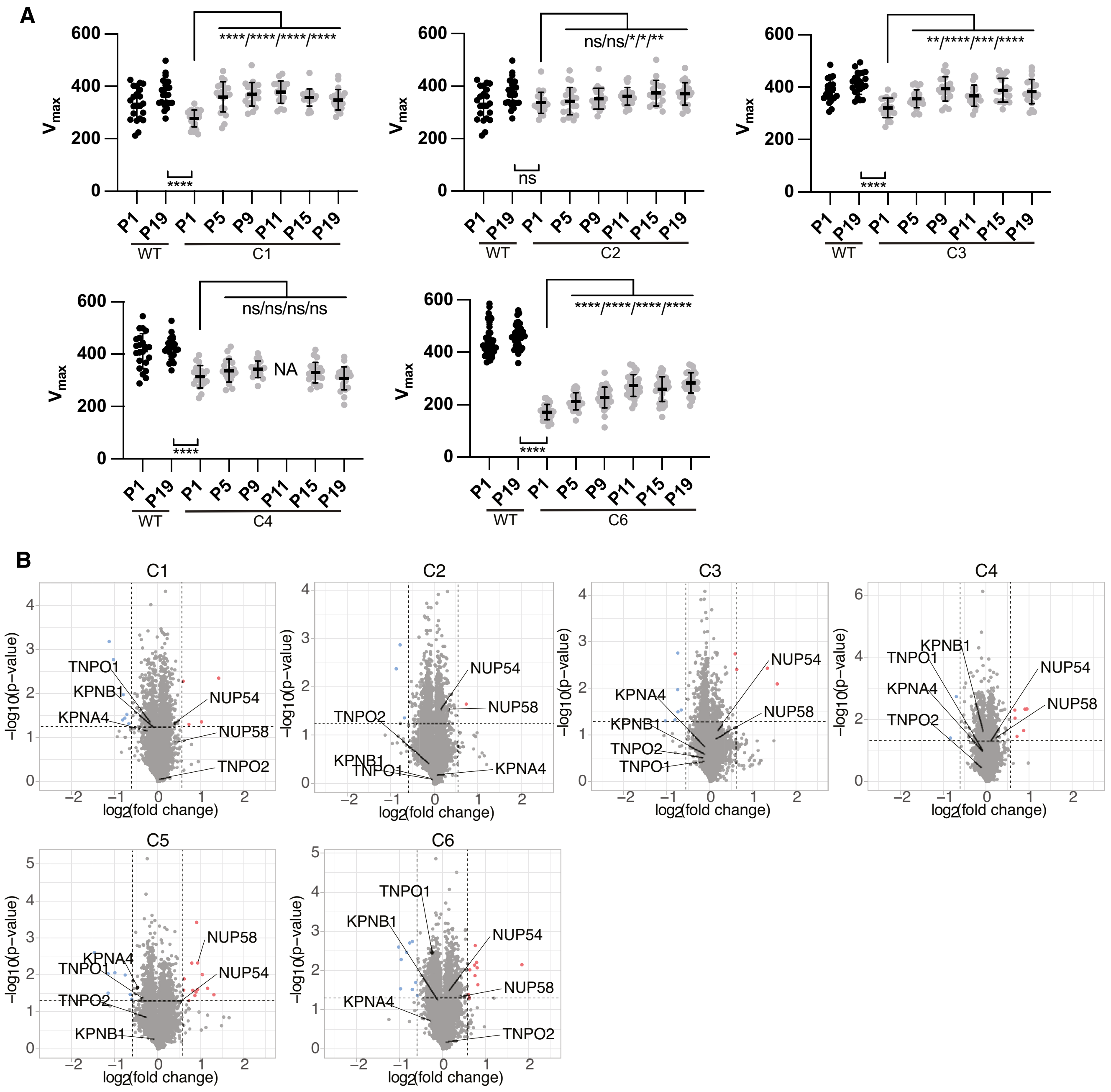

Figure EV4. Long-term fitness recovery and proteome changes in HAP1 mutant clones.

A Growth rates of passaged mutant HAP1 cell lines and matched controls. WT cell lines were analyzed at P1 and P19 only. NUP58 mutant clones were analyzed at the indicated passage numbers. Each dot represents a technical replicate (n = 22) of one experimental replicate performed. Error bars indicate mean with SD (Welch's t-test; ns = non-significant, **$P \leq 0.0030$, ****$P \leq 0.00001$).

B Volcano plots reporting the level of protein in each clone at P19 (C1 to C6) as a ratio relative to cell lines at P1. Red and blue dots indicate upregulated and down-regulated genes/proteins, respectively. Select relevant genes/proteins are labeled. For transcriptome = P-value < 0.01, fold-change > 2; for proteome = P-value < 0.05, fold-change > 1.5.

© 2021 The Authors

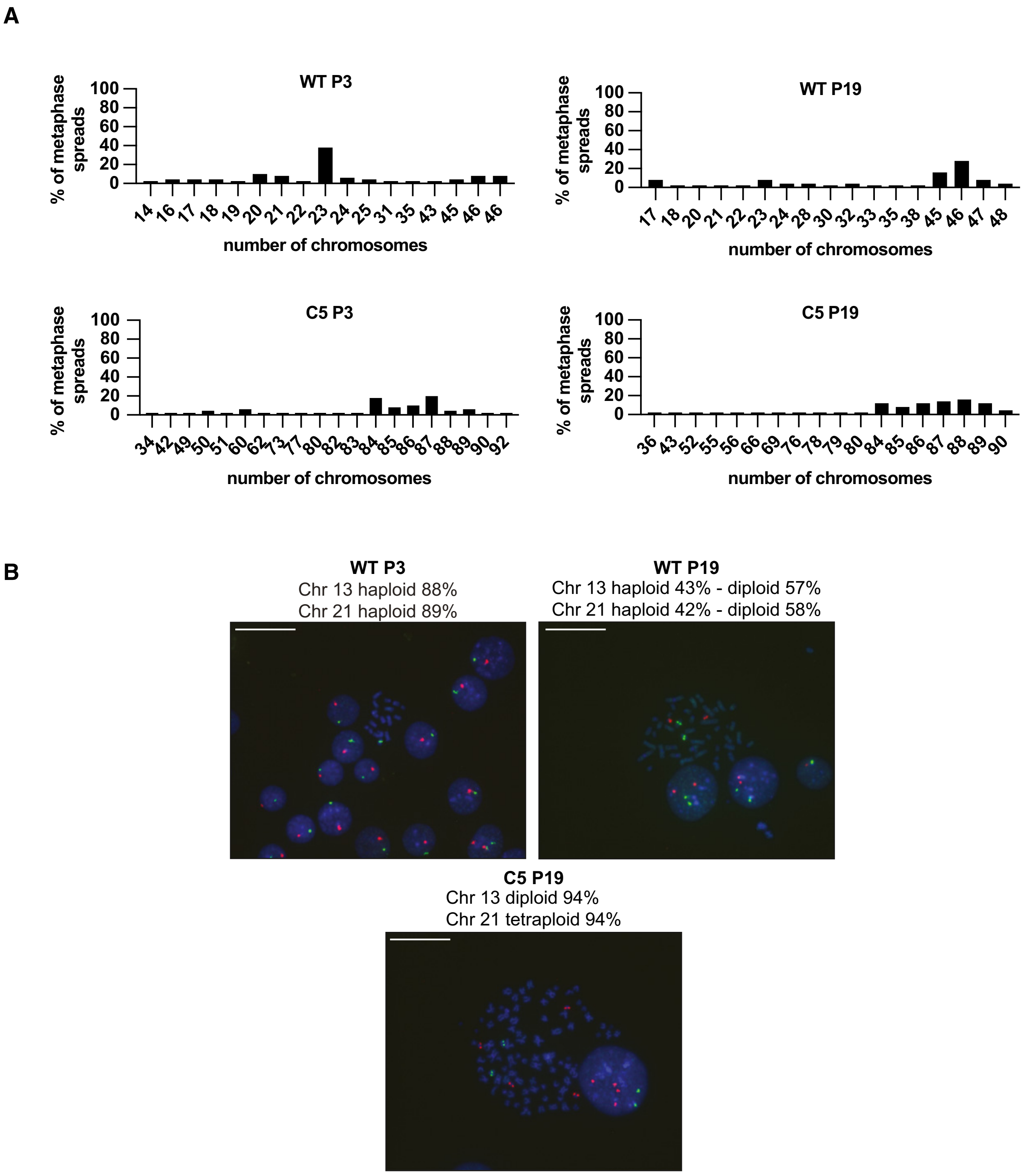

Figure EV5. Karyotype analysis for mutant clone C5.

A Chromosome counts from metaphase spreads of HAP1 control lines (WT P3–WT P19) and mutant C5 at P19 (n = 50).

B Representative images of chromosome-specific FISH hybridization for HAP1 control cell lines (WT P3–WT P19) and mutant C5 at P19. Green label Chr 13; orange label Chr 21. Scale bar, 31.3 μm.

 © 2021 The Authors
