# Supplementary figures and images for: Non‐genetic and genetic rewiring underlie adaptation to hypomorphic alleles of an essential gene
Source: EMBO J. 2021 Sep 15;40(21):e107839. doi: 10.15252/embj.2021107839 (PMC8561638; doi:10.15252/embj.2021107839)

Figure Appendix 2C

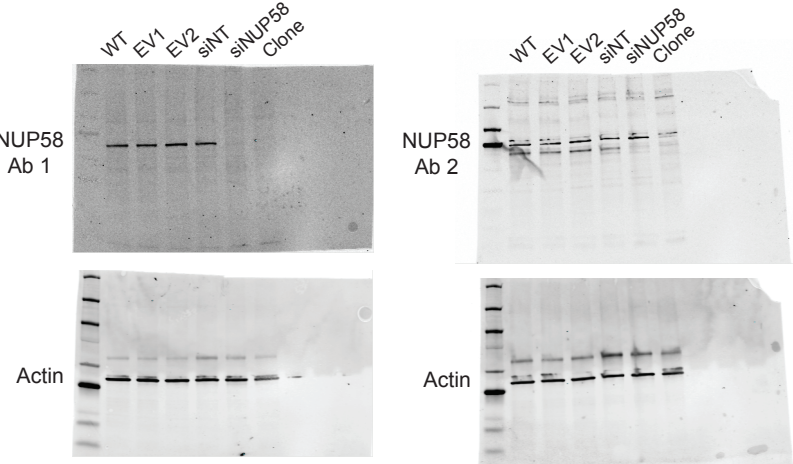

Supplement: Supplementary file 3 — Source Data for Appendix [file EMBJ-40-e107839-s005.zip › source_data_Appendix/source_data_appendix2C.pdf]

Figure Appendix 6

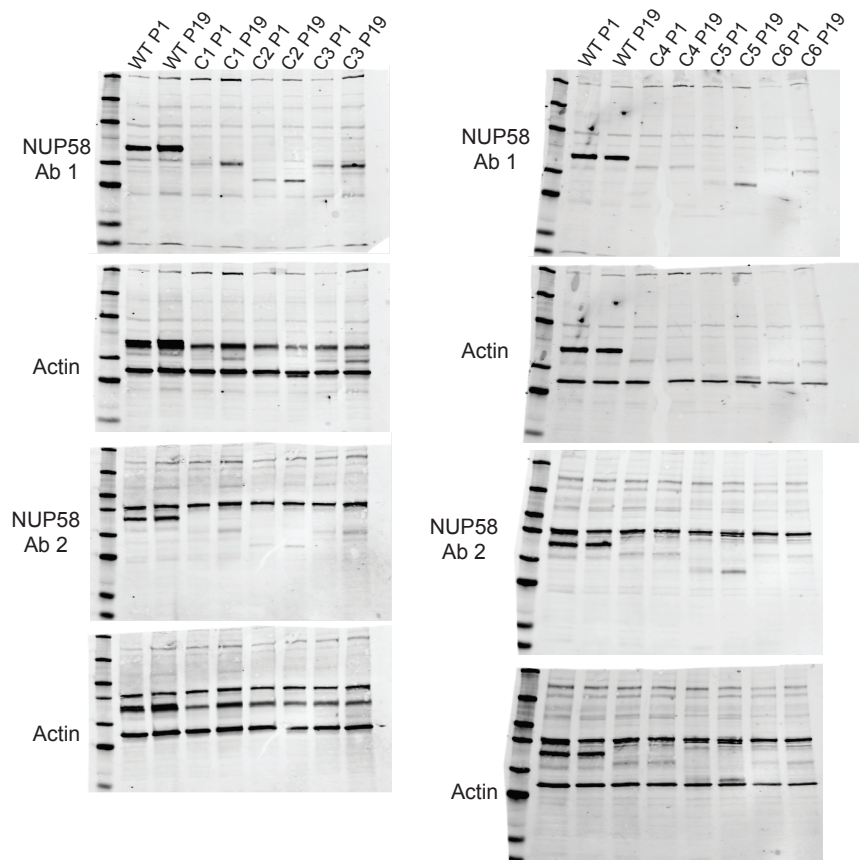

Supplement: Supplementary file 3 — Source Data for Appendix [file EMBJ-40-e107839-s005.zip › source_data_Appendix/source_data_Appendix_Fig6.pdf]

Figure 2E

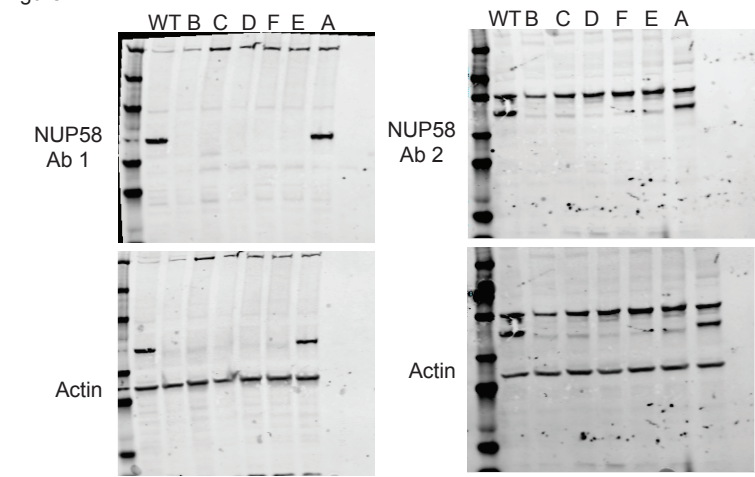

Supplement: Supplementary file 4 — Source Data for Figure 2 [file EMBJ-40-e107839-s002.zip › source_data_fig2E.pdf]

Figure 4B

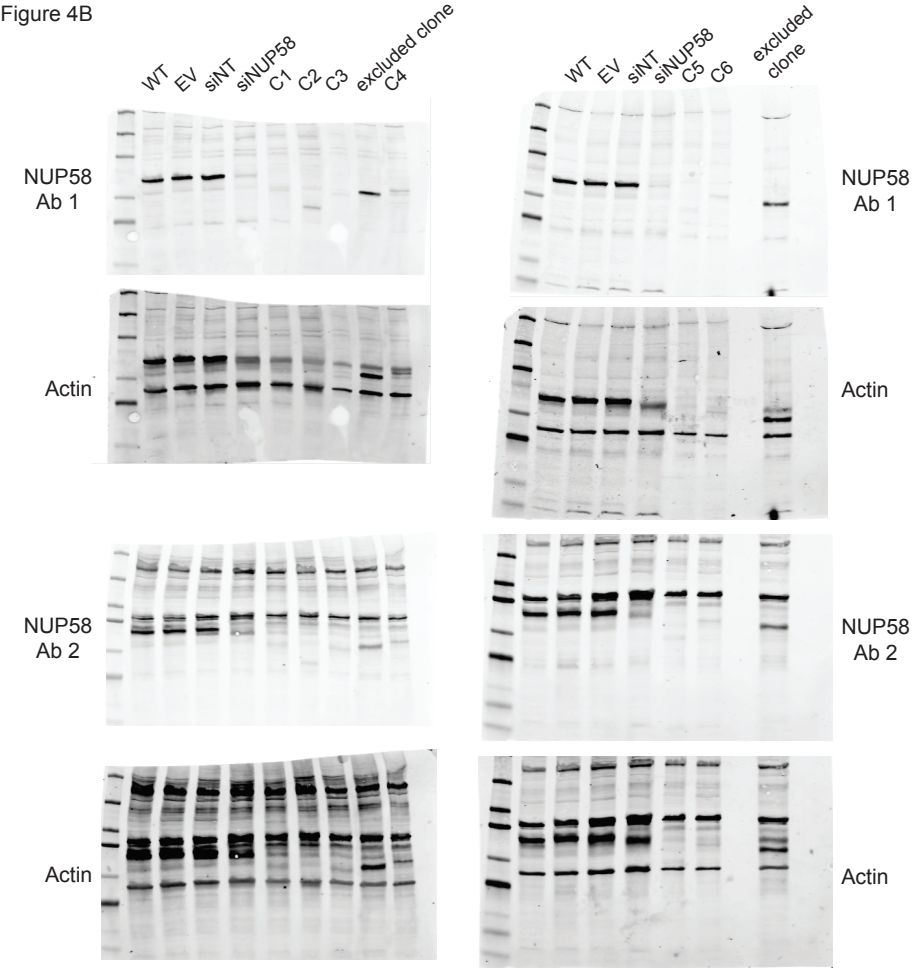

Supplement: Supplementary file 5 — Source Data for Figure 4 [file EMBJ-40-e107839-s006.zip › source_data_fig4B.pdf]

Figure 6E

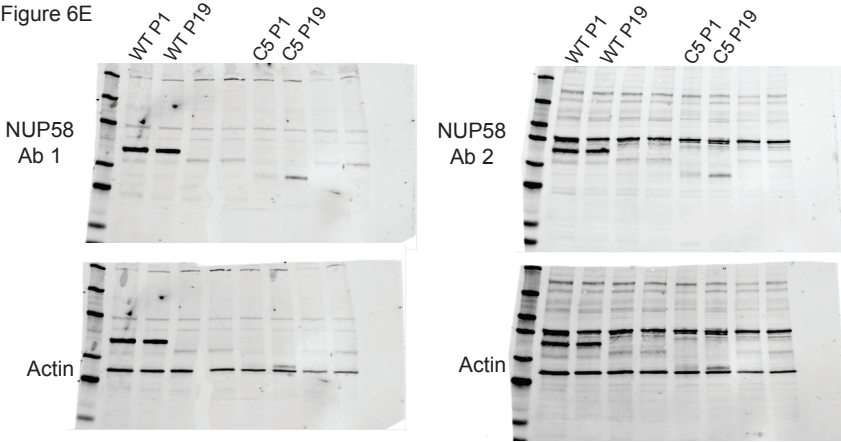

For unlabeled clones refer to  
Source Data Appendix Fig 6

Supplement: Supplementary file 6 — Source Data for Figure 6 [file EMBJ-40-e107839-s007.zip › source_data_fig6E.pdf]
