# Supplementary material for: Non‐genetic and genetic rewiring underlie adaptation to hypomorphic alleles of an essential gene
Source: EMBO J. 2021 Sep 15;40(21):e107839. doi: 10.15252/embj.2021107839 (PMC8561638; doi:10.15252/embj.2021107839)
Supplement: Supplementary file 7 [file EMBJ-40-e107839-s003.pdf]

Non-genetic and genetic rewiring underlie adaptation to hypomorphic alleles of an essential gene

Altea Targa, Katherine Larrimore, Cheng Kit Wong, Yu Lin Chong, Ronald Fung, Joseph Lee, Hyungwon Choi, and Giulia Rancati
DOI: 10.15252/embj.2021107839

Corresponding author(s): Altea Targa (alte91a@gmail.com)

Review Timeline:

Transfer from Review Commons:	27th Jan 21
Editorial Decision:	1st Feb 21
Revision Received:	2nd Jun 21
Editorial Decision:	12th Jul 21
Revision Received:	5th Aug 21
Accepted:	23rd Aug 21

Editor: Stefanie Boehm

Transaction Report: This manuscript was transferred to EMBO Reports following peer review at Review Commons.

(Note: With the exception of the correction of typographical or spelling errors that could be a source of ambiguity, letters and reports are not edited. Depending on transfer agreements, referee reports obtained elsewhere may or may not be included in this compilation. Referee reports are anonymous unless the Referee chooses to sign their reports.)

Review #1

1. How much time do you estimate the authors will need to complete the suggested revisions:

Estimated time to Complete Revisions (Required)

(Decision Recommendation)

Between 1 and 3 months

2. Evidence, reproducibility and clarity:

Evidence, reproducibility and clarity (Required)

In this paper, Targa et al investigate how cultured human cells adapt to a genetic perturbation. They investigate the role of both cellular plasticity (non-genetic adaptation processes) and genetic mutations in the adaptation to hypomorphic alleles of NUP58. The hypomorphic alleles encode truncated proteins with a low expression level, that all cause a fitness defect compared to "wild-type" cells. The magnitude of this growth defect appears to correlate with the extend of impairment of nuclear import. The authors describe transcriptional changes that are associated with early adaptation, and a genetic change associated with long-term adaptation.

I found this a very interesting paper, that takes experimental evolution from microorganisms to mammalian cells. The paper is well-written, all data are clearly presented, the figures are easy to interpret, and I have no concerns about statistics or replication. However, some points were not entirely clear to me, and I do have a few comments and questions about the authors' conclusions.

Major comments:

1.At several points in the text, the authors state that early adaptation occurred due to phenotypic plasticity by karyopherin overexpression. I have a few concerns about this statement:

a.The performed experiments do not exclude a role for genetic variation. It is possible that these 6 clones only survived the NUP58 mutations because they had a specific genetic variant elsewhere in the genome. The authors briefly mention in the text that they performed whole exome sequencing on the 6 clones. At which time point was this, and did the authors identify any potentially adaptive mutations? Alternatively, could they use their RNAseq data to exclude the possibility of adaptive coding variants at the "early" timepoint?

b.RNAseq and proteomics data - I am not getting a clear picture of the general trends that were found in these data. The authors focus on the NPC-genes that are down-regulated and the karyopherins that are upregulated in the early response, but this seems a bit cherry-picking in the absence of a more general overview of the

data. Are these some of the strongest / main signals in the data? How many genes were up/down regulated? Were there functional enrichments among these gene sets?

c.The authors assume that the expression changes they see for the karyopherins are adaptive. However, as it is unclear how specific these changes are (see previous point), upregulation of the karyopherins could just be part of a general stress response. The authors do not directly show that karyopherin overexpression improves fitness of the NUP58 mutant cells.

2.Figure 6B, EV5 - The data for C5 P1 are missing. These data are crucial for proving that the NUP58 amplification was not present at P1, but occurred during adaptation.

Minor comments:

1.At several points in the text, the authors discriminate "early adaptation" from "long-term adaptation". However, after reading the main text and methods, it remains unclear to me what their definition of "early" or "P1" is. I doubt that this is really directly after transfection, as it takes some time for genome editing to take place and for the NUP58 protein to be degraded? How many days or generations post infection is this?

2.Figure 1C - I find it interesting that no viable clones were obtained for NUP85, some for NUP58, and some more for NUP153. Do the authors have some suggestions to why they see these differences in number of surviving clones? Are the domains targeted in NUP85 more "essential"? Or does it depend on whether an exon can be skipped without introducing a frameshift? Would targeting an exon of N x 3 nucleotides be a general strategy for making hypomorphic mutant alleles? I think a bit of discussion on this would be interesting for readers who may want to make hypomorphic alleles of their gene of interest.

3.I am a little surprised that the authors do not cite their own work in yeast (Liu et al., 2016, Cell) where they have shown adaptation of NUP-mutants by aneuploidies. I think a short discussion on the differences between adaptation mechanisms between yeast and HAP1 cells would be interesting.

4.Figure EV3B, S3 - The distributions of the C3 and C5 data look strange, there appears to be a problem with the normalization of these samples.

5.The RNAseq, WES, and proteomics data should be included in the supplement or uploaded to a public database.

6.Figure EV1E - the actin blot is missing for NUP58

3. Significance:

Significance (Required)

In my opinion, the two main advances of this paper are:

1.Studying the spontaneous adaptation of human cells to genetic perturbations. To my knowledge, this paper is (one of) the first to describe adaptive mechanisms to genetic perturbations in mammalian cells. It remains to be seen if the mechanisms that the authors describe for NUP58 hold for other genes, but I think this is an important first step. I suspect that many similar studies will follow in the next years, that apply a similar methodology to different genes, cell lines, and types of alleles.

2.Using CRISPR-Cas9 to create "stable" hypomorphic cell lines. Creating new hypomorphic mutant alleles to study a gene of interest is well-established in many model organisms, but its use is still quite limited in mammalian cell culture, even though it has several advantages over for example tetracyclin-repressible or loxP alleles (no need for drugs, all cells have same knockdown effect). The authors cite one example using mouse cell lines (Challa et al., 2016).

Audience:
This paper will be of interest to a broad range of geneticists.

My expertise:
Yeast genomics, human genetics, CRISPR-technology development.

Review #2

1. How much time do you estimate the authors will need to complete the suggested revisions:

Estimated time to Complete Revisions (Required)

(Decision Recommendation)

Cannot tell / Not applicable

2. Evidence, reproducibility and clarity:

Evidence, reproducibility and clarity (Required)

The authors used CRISPR-Cas9 to generate hypomorphic alleles of NUP58, NUP153, and NUP85 in the haploid leukemia cell line HAP1. They obtained a few viable clones for NUP58 and NUP153, no viable clones for NUP85, and concluded that all three nucleoporins are essential for viability of HAP1 cells. Further characterization of the NUP58 clones revealed that they contained hypomorphic alleles with indel mutations and premature termination codons (PTCs) in two of the clones. All clones showed impaired growth rates, two clones showed significant import defects and one clone bimodal import characteristics. The reduced fitness of NUP58 hypomorphic alleles was confirmed in HCT116 cells by inserting a PTC. The authors next performed RNAseq and quantitative mass spec and found that transcript and protein levels of the truncated NUP58 were reduced compared to control cells, likely due to reduced stability of the mRNA. Similarly, other nucleoporins are reduced, whereas nuclear transport receptors (NTRs), in particular TNPO1, TNPO2, and KPNA4, are upregulated. To study the long-term effect of the hypomorphic NUP58, one clone (clone 5) was analyzed in long-term adaptation experiments (40 duplications, 20 passages). The authors found that fitness and nuclear import of this clone was restored, that expression of truncated NUP58 and other nucleoporins was stabilized, and that expression of NTRs was downregulated, as compared to early passage numbers. They conclude that NUP58 function was restored by long-term adaptation.

Specific comments:

1.Page numbers are missing!

2.Figure 2: the author studied nuclear import of an NLS-3xmCherry construct and observed for two clones significant nuclear import defects. Please provide representative images, not only the statistics.

3.Figure 4: RNAseq and quantitative mass-spec analysis of early adaptation to loss of NUP58 function revealed that NTRs (TNPO1, TNPO2, KPNA4) are upregulated and the authors speculate that by this is to support nuclear import. How does this match with the above observation that nuclear import is significantly reduced, especially in clone 5 and 6? Please provide data for TNPO1- and TNPO2- as well as KPNA4-dependent cargos. Also, KPNB1 is upregulated, how does this match with the observed import defects of the KPNB1-dependent reporter construct?

4.Figure 4: how do the authors explain that NUP62, the major binding partner of NUP58, is down-regulated on the transcript level, but upregulated on protein level? Part of the adaptation effects, in particular in the long-term adaptation experiments, might indeed arise from the fact that NUP62 is compensating for the loss of NUP58. NUP62 upregulation during long-term adaption (Fig. 5C) is not as strong as for NUP58 and NUP54, the other complex partner, but this could be due to the fact that NUP62 is immediately upregulated upon loss of NUP58.

5.Figure 5: the statistics presented in Fig. 5A and 5B are not clear. The authors claim that the fitness of clone 5 is restored to WT levels, but the data do not show this. The fitness might be increased compared to P1, but is still reduced compared to WT. It is not clear what was used as reference in the statistical analysis: it seems that values of P5-P19 were compared to P1 values of clone 5, but not to WT. Please provide a proper and clear statistical analysis. Also, please provide images that show the restored nuclear import capacity of clone 5 upon long-term adaptation.

6.Figure 5: the authors show that upon long-term culturing the localization of NUP58 at NPCs is restored/increased and conclude that the mutant protein is indeed functional. Functionality cannot be concluded from proper localization, a proper localized protein can be non-functional. NUP58 localization to NPCs is mediated by its coiled-coil domain, function by the FG domain. The truncated NUP58 in clone 5 (and the corresponding HCT116 variant) maintained the coiled-coil domain, but lack the C-terminal FG domain. How can this protein be functional? Please provide data that show functionality of the protein. For the overall conclusion it is important to show that it is indeed NUP58 function that has been restored and that the adaptation is not due to the fact that other nucleoporins compensate loss of NUP58 function, for example NUP62.

3. Significance:

Significance (Required)

see above

Review #3

1. How much time do you estimate the authors will need to complete the suggested revisions:

Estimated time to Complete Revisions (Required)

(Decision Recommendation)

Between 1 and 3 months

2. Evidence, reproducibility and clarity:

Evidence, reproducibility and clarity (Required)

Summary:

In the current manuscript, Targa and co-workers set out to explore the potential molecular mechanisms by which a human chronic myeloid leukemia cell line could compensate for the genetic perturbation of an essential gene. In short, they investigated the initial physiological response to this perturbation then also performed an adaptive laboratory evolution (ALE) experiment to shed light on the possible evolutionary mechanisms that could mitigate the harmful effects. Briefly, they created conditional alleles of a nuclear pore protein complex member, and as a consequence generated truncated proteins with low abundance. They found that functionally related genes got upregulated as a physiological response, and this paved the way for the emergence of genomic mutations that compensated the initial fitness defect. This could potentially mean that inhibiting these early transcriptional changes during targeted drug therapy could be a useful strategy to limit the development of resistance towards the targeting agent.

Major comments:

I felt that the manuscript would require some additional analyses and extension of the text to fill in some gaps in order to provide some further support for their conclusions and also to increase clarity. See my detailed comments below.

1.The authors selected several nuclear pore proteins, all essential, and created conditional alleles. They also targeted a non-essential gene as a control. Maybe I am missing something or I have limited knowledge in working with human cell lines, but it was strange to read that even after targeting a non-essential gene, a 40 % drop in clone viability was observed according to Fig1C. Could this be a simple consequence of the potential off-target effect of CRISPR or did other unrelated factors affect viability? May it be the case that the transcriptome profile of the ancestor lines partly reflect a physiological response to potential off-target effects? Have the authors considered such possibilities? On a related note, Fig 1C shows relative viability, so basically, we don't have any information for the viability of cells transfected with a vector that lacks a gRNA.

2.The Authors have used 6 clones with perturbed NUP58 gene for further in-depth analysis. All of the clones showed a significant fitness decrease based on Fig 2C with a somewhat similar effect size for most of them. However, the cargo efficacy experiment demonstrated that some of the clones had WT-like nuclear transport. The Authors proposed a possible explanation for that in the form of rapid evolution during the transfection/selection steps of the reporter construct. This could have been possibly tested by re-measuring the fitness of the clones carrying the reporter construct and showing WT-like nuclear transport and thereby demonstrating that they no longer have any large fitness defect. By the way, exactly how many generations

are we talking about right here during this rapid evolution? Would we expect genetic or transcriptional changes during this presumably short period of time?

3.According to Fig EV2A, the total NUP58 read count (from the RNA-sequencing dataset) was decreased in all clones (interpreted as destabilized mRNA), however a big variation was apparent across the clones (ranging from ~0.4 to ~0.8 relative expression), an observation that was not visible in Fig EV2B, where protein levels were plotted. It would be helpful to have an interpretation for this difference in the manuscript (e.g. post-transcriptional regulation?).

4.It might be informative to investigate if there is any correlation between expression level/protein level/transport efficacy and fitness across the lines (both ancestor and evolved).

5.Expression analysis of the different clones revealed major differences in the transcriptome profile of different clones. For example, clone 3 and clone 5 display a totally different response to the perturbation of NUP58, also suggesting different off-target effects across the clones (ie. see major comment 1). The authors might consider trying to cluster the clones according to their profile, in addition to Fig App S3, where the transcripts were clustered. By doing that, clones with similar profiles would be more visible. Also by having that we might be able to better understand the phenotypic differences across the lines.

6.The Authors report in the manuscript that NPC components are generally downregulated. Maybe I am missing something, but I failed to see that in Fig EV3A, where mRNA levels were plotted in each clone relative to the wild-type control line. Out of the NPC components, only the NUP54 are marked besides NUP58 (ie. the perturbed gene), and it does not seem to reach either the fold-change or the p-value cutoff, at least in the case of transcript levels. The protein level of NUP54 seems to be more likely in accordance with the text (and with Fig4), although some of the clones still don't seem to have a lower NUP54 protein level. The same applies to the upregulated karyopherins that do not seem to be upregulated in all of the clones (even at protein level). It would be helpful if the Authors could elaborate on this, and explain the rationale for the calculation of the average instead of the median value of the clones on Fig 4A-B, which would have been less sensitive to an outlier clone. Although I am still not sure if calculating summary statistics (mean or median of 6 clones) of expression levels is the right way to make any inferences on the response in general as the clones have different fitness/cargo efficacy. I also recommend to display these functional groups with a different color (or shape) on Fig EV3A-B for clarity. Alternatively, make another plot that shows only these functional categories. Do we really see them as being significantly up/downregulated? Do we have a significant enrichment of these functional categories among the up/downregulated genes? This can be easily tested by performing Fisher's exact test to probe whether being/not being in the functional category shows any association with being/not being in the set of up/downregulated genes.

7.Upregulation of karyopherins (transport receptors) seems to be an early adaptive response to the perturbed NUP58 function. Would it be possible to test this hypothesis by artificial overexpression of such a protein? I also wonder if there is an expression limit for a nuclear membrane protein without having any toxic effect (ie. dosage sensitivity). The Authors might also consider finding some further support for their hypothesis by looking for synthetic sick/lethal interaction between the yeast homologs of nuclear pore proteins and karyopherins based on previous genetic interaction screens in baker's yeast.

8.The evolutionary experiment lasted for 20 passages, ie. 40 generations, which seems to be a quite short evolutionary experiment, compared to those conducted in single-celled microorganisms, where hundreds of generations are reached. Although I am not an expert in evolutionary studies in human cell lines, this is all the more intriguing if we consider that the mutational supply depends on the mutational rate and population size, both of which I would expect to be lower in human cells compared to bacteria/yeast. Since we don't have any information on these factors in the current setup, a solid explanation and a more detailed methods

section (currently there is no such) for the evolutionary experiment would be required.

9.Fig EV4A shows the fitness of the 6 clones before and after evolution. I made several observations by looking at this figure. First, C2 seems to be an outlier, as it has no significant fitness cost at P1 compared to the WT. Could this be due to the karyopherin overexpression? Does this clone have a higher expression level than the rest of the clones? Second, clone 4 did not improve its fitness, as a matter of fact, it seems that there is a fitness decline at the end of the experiment. Is there any explanation for that? Could it be due to lower initial upregulation of karyopherins? Third, it is hard to tell, whether there is any main difference in the relative fitness improvement among the rest of the clones. This can be easily tested if the Authors normalize the fitness of each passage with the first one (or maybe even to the passage 0, ie fitness displayed on Fig 2C?).

10.The Authors chose C5 for in-depth analysis, even though they do not provide any criteria for that. They found that the NPC was stabilized, demonstrated by increased efficacy of nuclear transport (Fig5B) and increased NUP58 protein level (Fig5D). As a possible mechanism, they found a strong focal amplification (demonstrated by FISH) of the NUP58 instead of chromosome aneuploidy. However, this was not reflected in protein level, as it still seems to have a significantly lower protein level than the non-truncated NUP58 level in the WT (Fig5D), although the discussion claims it otherwise: "restoration of mutant protein expression to wild-type levels correlated with a significant fitness gain". Nevertheless, the results suggest there is a large contribution of posttranscriptional regulation that keeps the mutant protein level under the wild-type. This is quite intriguing as without such a regulation the strong amplification of NUP58 would also imply a stoichiometric imbalance among the members of the NUP complex which should subsequently result in a fitness loss as well. I would appreciate any comment on this issue.

Minor comments:

1.The manuscript is well written. The figures are well designed and provide clear messages.

2.Fig 5B is not referred to in the text.

3.The method(s) to correct the p-values for multiple testing is not mentioned in the text, please add it accordingly.

4.Prior work(s) that investigated the compensability of essential gene perturbations using baker's yeast as a model should be cited in this manuscript.

3. Significance:

Significance (Required)

The subject and the general plan of the present study are well chosen. It was stimulating to read an ALE experiment that used a human cell line instead of the commonly used single-celled microorganisms like yeast and bacteria. I believe that such a work could potentially pique the interest of anyone in the broad field of evolutionary biology and potentially also beyond that. Similar works like this current study could help in better understanding whether the general principles we learned about compensatory mechanisms in the simpler model organisms can also be applied across different kingdoms of Eukaryotes. Since I work in the field of yeast biology and I am not an expert of human-related techniques/protein techniques (eg. Western blot) I might found some of the methods/results section inappropriate. If they follow established protocols/concepts, please ignore my corresponding comments.

1st Editorial Decision 1st Feb 2021

Dear Dr. Rancati,

Thank you for transferring your manuscript from Review Commons to The EMBO Journal. I have now carefully read your study, as well as looked at the review reports and your response, and discussed everything with the other members of the editorial team. Overall, we have decided to invite a revision of the manuscript at the EMBO Journal.

We appreciate that you have already addressed some of the initial referee concerns in the version submitted and provided a preliminary point-by-point response. The experiments, analyses and additional discussion proposed in this response should be incorporated into a revised version. Here, it will be especially important to fully address the key issues before we can consider the study further for publication. In particular, further defining the events of "early adaptation" and adding proof that adaptive transcriptional plasticity occurs (ref#1- major point 1a-c; ref#3- point 2, 7, 9). In addition, the outcome of long-term culture for NUP58 function should be investigated in further detail and carefully discussed, in particular given the question referee #2 raises regarding protein determinants required for function vs. sub-cellular localization (ref#2- point 6; related to ref#3- point 10). Please also carefully discuss the more general statements regarding potential implications for drug resistance, also taking into account the potential differences between an engineered mutant, which may affect several biological processes (ref#1-1b response), and a targeted drug. Finally, please also revise the manuscript to ensure that the Material and Methods section contains all necessary information in full detail and a Data Availability section (please see below for more information).

Please be aware that it is our policy to allow only a single round of major revision. We realize that addressing all referee points fully, in particular the key issues, will require a substantial revision and additional analyses with potentially unclear outcomes. We are aware that lab work worldwide is currently affected by the COVID-19/SARS-CoV-2 pandemic and that an experimental revision may be delayed, and we can extend the revision time when needed, and we have extended our 'scooping protection policy' to cover the period required for a full revision. However, we will require strong support from all referees on the revised version of the manuscript in order to consider it further for publication at EMBO Journal. If you do not want to commit to such a major revision or forsee inevitable problems in doing so, please contact us to discuss potential alternatives as soon as possible.

Please also feel free to contact me should you have any other further questions. Thank you for the opportunity to consider your work for publication.

Kind regards,

Stefanie Boehm

Stefanie Boehm
Editor
The EMBO Journal

Response to Reviewers:

Reviewer #1 (Evidence, reproducibility and clarity (Required)):

In this paper, Targa et al investigate how cultured human cells adapt to a genetic perturbation. They investigate the role of both cellular plasticity (non-genetic adaptation processes) and genetic mutations in the adaptation to hypomorphic alleles of NUP58. The hypomorphic alleles encode truncated proteins with a low expression level, that all cause a fitness defect compared to "wild-type" cells. The magnitude of this growth defect appears to correlate with the extend of impairment of nuclear import. The authors describe transcriptional changes that are associated with early adaptation, and a genetic change associated with long-term adaptation.

I found this a very interesting paper, that takes experimental evolution from microorganisms to mammalian cells. The paper is well-written, all data are clearly presented, the figures are easy to interpret, and I have no concerns about statistics or replication. However, some points were not entirely clear to me, and I do have a few comments and questions about the authors' conclusions.

R: We thank the reviewer for finding our manuscript "very interesting" and "well-written" and for acknowledging that it opens the field of experimental evolution to mammalian cells.

Major comments:

1.At several points in the text, the authors state that early adaptation occurred due to phenotypic plasticity by karyopherin overexpression. I have a few concerns about this statement:

a.The performed experiments do not exclude a role for genetic variation. It is possible that these 6 clones only survived the NUP58 mutations because they had a specific genetic variant elsewhere in the genome. The authors briefly mention in the text that they performed whole exome sequencing on the 6 clones. At which time point was this, and did the authors identify any potentially adaptive mutations? Alternatively, could they use their RNAseq data to exclude the possibility of adaptive coding variants at the "early" timepoint?

R: The exome sequencing samples were submitted from the exact same pool of cells that were used for RNAseq and MS analyses and it is the earliest possible passage after the generation of the clones. Therefore, to respond to the reviewer's comment, we reanalyzed the exome sequencing datasets for all 6 clones in search for mutations that are common in all clones and could support viability in presence of NUP58 mutations. As shown in new Appendix Figure S3A, the only two exome mutations that are present in all clones are mutations in NUP58 and in AASDH, a beta-alanine activating enzyme. We think that such mutation is unlikely to be adaptive in response to NUP58 inactivation for two reasons. First of all, there is no evidence in the literature that alanine metabolism is implicated in nuclear trafficking. Secondly, we also noted that the AASDH mutation map in an A-rich and difficult to sequence region, suggesting that it might be a false positive. We also checked mutations that are present in at least 3/5 clones and found 15 of them.

As for AASDH, the mutations map in difficult to sequence regions and are therefore likely false positive (Appendix Figure S3A).

Top sum up, analysis of exome sequencing did not find evidence of common mutations that could be adaptive in response to NUP58 inactivation. Moreover, as discussed in response to point 1C, we now showed evidence that karyopherin overexpression is sufficient to increase the viability of freshly generated NUP58 mutant clones. Taken together, these evidences support our hypothesis that initial viability in response to NUP58 inactivation is not driven by genetic variation but by phenotypic plasticity and specifically by karyopherin overexpression.

The text has been revised accordingly.

b.RNAseq and proteomics data - I am not getting a clear picture of the general trends that were found in these data. The authors focus on the NPC-genes that are down-regulated and the karyopherins that are upregulated in the early response, but this seems a bit cherry-picking in the absence of a more general overview of the data. Are these some of the strongest / main signals in the data? How many genes were up/down regulated? Were there functional enrichments among these gene sets?

R: We apologize for the lack of clarity on this point. The inactivation of the NPC has been shown to trigger differential expression of several hundred of developmental and transcription factor genes (Raices et al., 2017 Curr Opin Cell Biol; D'Angelo et al., 2018 Nucleus). As shown in Appendix Figure S3B also our dataset was dominated by these two classes of genes, making it difficult to differentiate between genes which expression change in response to lack of functional NPC vs genes which expression change as an adaptation to the loss of NPC function. To specifically looking at the latter set, we selected differentially expressed proteins with log2FC >0.2 which are known to genetically interact with the NPC in yeast (Costanzo et al., Science 2016). As shown in new Appendix Figure S3C, proteins involved in nuclear-cytoplasm transport and trafficking were differentially expressed, confirming our initial analysis shown in Figure 5A-B.

c.The authors assume that the expression changes they see for the karyopherins are adaptive. However, as it is unclear how specific these changes are (see previous point), upregulation of the karyopherins could just be part of a general stress response. The authors do not directly show that karyopherin overexpression improves fitness of the NUP58 mutant cells.

R: This is a great point. As shown in Figure 5A-5B, unpassaged early-derived NUP58 mutant clones upregulated the mRNA and protein expression of TNPO1-TNPO2-KPNB1-KPNA4 karyopherins. To test if karyopherin upregulation is sufficient to sustain early adaptation in response to NUP58 mutations, we needed to overexpress karyopherins. This experimental procedure was not trivial as, as mentioned by Reviewer #3, the overexpression of certain karyopherins, such as TNPO1, is toxic in both mammalian and bacterial cells. After trying different strategies, we successfully integrated KPNB1-KPNA4 under doxycycline-inducible promoter in a WT HAP1 cell line. As shown in new Figure 5C-D, induction of KPNB1-KPNA4 overexpression significantly increases the viability of freshly generated NUP58 mutant clones. We also noted that there is a slight increase in

the viability of NUP58 single cell-derived clones in absence of doxycycline treated. This is expected as the doxycycline expression system is documented to be leaky with reported basal expression of the transgenes in absence of doxycycline (Meyer-Ficca et al., 2004 Anal. Biochem., Pham et al., 2008 Biothechniques). This result is in agreement with our hypothesis that early adaptation to NUP58 inactivating mutations is driven by phenotypic plasticity and specifically by a transient transcriptional upregulation of karyopherins.

2.Figure 6B, EV5 - The data for C5 P1 are missing. These data are crucial for proving that the NUP58 amplification was not present at P1, but occurred during adaptation.

R: We apologize for not adding this important control. After receiving the Reviewers comments, we tried to revive C5 P1 to perform FISH, but it was unsuccessful and cells died during the process. We therefore ran qPCR analysis on C5 P1 and P19 as well as control using the gDNA used in the exome sequencing experiment. As shown in new Figure 7B, the genomic copy number of NUP58 increases in accordance with the increased expression of mutant NUP58 protein, shown by western blot now in Figure 6E and MS dataset (Figure 6D).

Minor comments:

1.At several points in the text, the authors discriminate "early adaptation" from "long-term adaptation". However, after reading the main text and methods, it remains unclear to me what their definition of "early" or "P1" is. I doubt that this is really directly after transfection, as it takes some time for genome editing to take place and for the NUP58 protein to be degraded? How many days or generations post infection is this?

R: We apologize for the confusion on this point. We added to Appendix Materials and Methods "Evolutionary experiment" to better describe how P1 was generated. In short, P1 was the earliest time point after NUP58 gene-editing and single cell seeding when we were able to collect enough cells to carry out exome sequencing, RNAseq and MS analyses as well as preserving cell aliquots. We calculated that we prepared roughly 15 X 10^6 cells to be able to perform such experiments. So if we assume no cell death, P1 would correspond to roughly 23 generations after the generation of the initial mutant single cell.

2.Figure 1C - I find it interesting that no viable clones were obtained for NUP85, some for NUP58, and some more for NUP153. Do the authors have some suggestions to why they see these differences in number of surviving clones? Are the domains targeted in NUP85 more "essential"? Or does it depend on whether an exon can be skipped without introducing a frameshift? Would targeting an exon of N x 3 nucleotides be a general strategy for making hypomorphic mutant alleles? I think a bit of discussion on this would be interesting for readers who may want to make hypomorphic alleles of their gene of interest.

R: We think this is a great point to discuss. For instance, the CRISPR/Cas9 targeted exon (exon 4) in NUP58 is a multiple of 3nt. Viable mutant clones (C2, C4, C5, C6) skipped the entire exon, avoided the PTC and generated a truncated mutant protein which retains some of its original functions. However, the exons that were targeted in NUP85 (exon 4

and 16) were not multiple of 3nt. As such, generation of a truncated mutant protein by exon skipping and generation of alternative isoforms is not possible. Since NUP85 is required for the overall stability and structure maintenance of the NPC, it is highly unlikely that cells will be able to survive in its complete absence.

3.I am a little surprised that the authors do not cite their own work in yeast (Liu et al., 2016, Cell) where they have shown adaptation of NUP-mutants by aneuploidies. I think a short discussion on the differences between adaptation mechanisms between yeast and HAP1 cells would be interesting.

R: We think this is a great suggestion and we will incorporate the reference into the discussion.

4.Figure EV3B, S3 - The distributions of the C3 and C5 data look strange, there appears to be a problem with the normalization of these samples.

R: We noted the distribution was not symmetrical in volcano plots displaying mass spectrometry data (EV3B and EV4B). We revised the normalization step for proteomics data and corrected the distribution in the revised manuscript.

5.The RNAseq, WES, and proteomics data should be included in the supplement or uploaded to a public database.

R: RNAseq, exome sequencing and proteomics data have now been uploaded to public databases (to be released to public upon manuscript acceptance) and referenced in the "Data Availability" section. Links for Reviewers access are provided below.

RNAseq GEO accession GSE161061:
Go to https://www.ncbi.nlm.nih.gov/geo/query/acc.cgi?acc=GSE161061
Enter token kzsrimuwfdgjxml ***into the box***

SRA exome sequencing data:
https://dataview.ncbi.nlm.nih.gov/object/PRJNA680690?reviewer=qfr48p1q56uvm8n6c8d0r4n2i5

PRIDE for proteomics
Username: reviewer_pxd022255@ebi.ac.uk
Password: qO4K6EIS

6.Figure EV1E - the actin blot is missing for NUP58

R: We apologize for the omission. We added the missing actin blot of Figure EV1E into the revised version.

Reviewer #1 (Significance (Required)):

In my opinion, the two main advances of this paper are:

1.Studying the spontaneous adaptation of human cells to genetic perturbations. To my knowledge, this paper is (one of) the first to describe adaptive mechanisms to genetic perturbations in mammalian cells. It remains to be seen if the mechanisms that the authors describe for NUP58 hold for other genes, but I think this is an important first step. I suspect that many similar studies will follow in the next years, that apply a similar methodology to different genes, cell lines, and types of alleles.

R: We thank the Reviewer for his/her support to our manuscript. This is indeed the first manuscript to dissect mechanisms of adaptation in human cells. Also, given the implications of dissecting adaptive evolution in mammalian cells, we are convinced that several other manuscripts using similar methodology will be published in the near future.

2.Using CRISPR-Cas9 to create "stable" hypomorphic cell lines. Creating new hypomorphic mutant alleles to study a gene of interest is well-established in many model organisms, but its use is still quite limited in mammalian cell culture, even though it has several advantages over for example tetracyclin-repressible or loxP alleles (no need for drugs, all cells have same knockdown effect). The authors cite one example using mouse cell lines (Challa et al., 2016).

R: We thank the Reviewer. We agree that the generation of hypomorphic alleles in mammalian cells using CRISPR/Cas9 is another novelty of the paper.

Audience:
This paper will be of interest to a broad range of geneticists.

My expertise:
Yeast genomics, human genetics, CRISPR-technology development.

Reviewer #2 (Evidence, reproducibility and clarity (Required)):

The authors used CRISPR-Cas9 to generate hypomorphic alleles of NUP58, NUP153, and NUP85 in the haploid leukemia cell line HAP1. They obtained a few viable clones for NUP58 and NUP153, no viable clones for NUP85, and concluded that all three nucleoporins are essential for viability of HAP1 cells. Further characterization of the NUP58 clones revealed that they contained hypomorphic alleles with indel mutations and premature termination codons (PTCs) in two of the clones. All clones showed impaired growth rates, two clones showed significant import defects and one clone bimodal import characteristics. The reduced fitness of NUP58 hypomorphic alleles was confirmed in HCT116 cells by inserting a PTC. The authors next performed RNAseq and quantitative mass spec and found that transcript and protein levels of the truncated NUP58 were reduced compared to control cells, likely due to reduced stability of the mRNA. Similarly, other nucleoporins are reduced, whereas nuclear transport receptors (NTRs), in particular TNPO1, TNPO2, and KPNA4, are upregulated. To study the long-term effect of the hypomorphic NUP58, one clone (clone 5) was analyzed in long-term adaptation

experiments (40 duplications, 20 passages). The authors found that fitness and nuclear import of this clone was restored, that expression of truncated NUP58 and other nucleoporins was stabilized, and that expression of NTRs was downregulated, as compared to early passage numbers. They conclude that NUP58 function was restored by long-term adaptation.

Specific comments:

1.Page numbers are missing!

R: We apologize for the inconvenience. We added both page and line numbers in the revised version.

2.Figure 2: the author studied nuclear import of an NLS-3xmCherry construct and observed for two clones significant nuclear import defects. Please provide representative images, not only the statistics.

R: We have now added the images of the cells in new Figure 2D.

3.Figure 4: RNAseq and quantitative mass-spec analysis of early adaptation to loss of NUP58 function revealed that NTRs (TNPO1, TNPO2, KPNA4) are upregulated and the authors speculate that by this is to support nuclear import. How does this match with the above observation that nuclear import is significantly reduced, especially in clone 5 and 6? Please provide data for TNPO1- and TNPO2- as well as KPNA4-dependent cargos. Also, KPNB1 is upregulated, how does this match with the observed import defects of the KPNB1-dependent reporter construct?

R: We thank the Reviewer for giving us the opportunity to better explain this point. The overexpression of karyopherins represent a short-term adaptation in response to the loss of NUP58 activity. While it allows cells to survive the insult, it is not optimal as cellular fitness is still majorly affected (growth rates, Figure 3B). As such, complete restoration of the nuclear import is not expected at this point. As cells are passaged, they are given the opportunity to acquire alternative adaptive mechanisms that further improve fitness and nuclear pore function. Indeed, as shown in Figure 6A, growth rate of C5 P19 is significantly improved compared to C5 P1 and concomitantly nuclear pore function is restored to wild-type levels (Figure 6B). At the mechanistic level, restoration of fitness and nuclear pore function is underlain not by overexpression of karyopherins, but by increased expression of mutant NUP58, as shown in Figure 6G. We will revise the discussion to better clarify this point.

As reported in new Figure 5D, overexpression of KPNA4 and KPNB1 is sufficient to increase viability of freshly generated NUP58 clones. Since KPNA4-KPNB1 activity is recapitulated by the use of NLS reporters, we decided to focus our effort using this reporter.

4.Figure 4: how do the authors explain that NUP62, the major binding partner of NUP58, is down-regulated on the transcript level, but upregulated on protein level? Part of the adaptation effects, in particular in the long-term adaptation experiments, might indeed arise from the fact that NUP62 is compensating for the loss of NUP58. NUP62 upregulation during long-term

adaption (Fig. 5C) is not as strong as for NUP58 and NUP54, the other complex partner, but this could be due to the fact that NUP62 is immediately upregulated upon loss of NUP58.

R: As showed in Chung et al., 2015 Science, the structures of NUP62 and NUP58 in metazoans are very different. Moreover, the stoichiometry of the Nup62 complex (NUP62• 54• 58) is specific and fixed to 1:1:1 and up to date no complex with 2 NUP62 subunits and one NUP54 have been characterized. We also note that, as reported by Boehmer et al., 2003 Proc Natl Acad Sci, proteins of the same NPC subunits are degraded after depletion of their interacting partners, or if the stoichiometry of the complex is otherwise out of balance, making it unlikely that the overexpression of NUP62 is adaptive. We note that while NUP54 has only be involved in the NUP62-54-58 complex, NUP62 also localizes at the cytoplasmic filaments, outside the channel. As such the modest NUP62 upregulation observed in Figure 6D (previous 5C) could be a consequence of an increased NUP62 cytoplasmic filament localization.

5.Figure 5: the statistics presented in Fig. 5A and 5B are not clear. The authors claim that the fitness of clone 5 is restored to WT levels, but the data do not show this. The fitness might be increased compared to P1, but is still reduced compared to WT. It is not clear what was used as reference in the statistical analysis: it seems that values of P5-P19 were compared to P1 values of clone 5, but not to WT. Please provide a proper and clear statistical analysis. Also, please provide images that show the restored nuclear import capacity of clone 5 upon long-term adaptation.

R: We apologize if the analysis was not clear. In new Figure 6A (unrevised 5A), all samples were compared to WT P1, whereas in new Figure 6B (unrevised 5B), C5 P3 and P19 were compared to C5 P1. We amended the figures and figure legends to better describe the comparisons made. Also, we agree with the reviewer that while C5 improves cellular fitness, it does not restore its growth rate to the control level. We amended the text accordingly. We also added representative images of the NLS-cherry nuclear import of C5 P1 and P19.

6.Figure 5: the authors show that upon long-term culturing the localization of NUP58 at NPCs is restored/increased and conclude that the mutant protein is indeed functional. Functionality cannot be concluded from proper localization, a proper localized protein can be non-functional. NUP58 localization to NPCs is mediated by its coiled-coil domain, function by the FG domain. The truncated NUP58 in clone 5 (and the corresponding HCT116 variant) maintained the coiled-coil domain, but lack the C-terminal FG domain. How can this protein be functional? Please provide data that show functionality of the protein. For the overall conclusion it is important to show that it is indeed NUP58 function that has been restored and that the adaptation is not due to the fact that other nucleoporins compensate loss of NUP58 function, for example NUP62.

R: We agree with the Reviewer this is a crucial point. To experimentally show that long-term adaptation in response to NUP58 mutations is driven by upregulation of NUP58 mutant form lacking exon #4, #14-16, we generated wild-type HAP1 cell line stably expressing the NUP58 mutant isoform. These cells were subjected to CRISPR/Cas9 gene editing targeting the native form of NUP58. Specifically, we concomitantly targeted exon 4 and exon 14, which are only present in the endogenous NUP58 but not in the NUP58 mutant form and single-cell plated the cell population. As shown in new Figure 6G and in

accordance with our hypothesis, cells overexpressing mutant NUP58 significantly formed more viable colonies than wild-type control after gene editing of the native NUP58. This experiment supports our hypothesis that overexpression of mutant NUP58 is functional and support NPC activity.

Reviewer #2 (Significance (Required)):

see above

Reviewer #3 (Evidence, reproducibility and clarity (Required)):

Summary:

In the current manuscript, Targa and co-workers set out to explore the potential molecular mechanisms by which a human chronic myeloid leukemia cell line could compensate for the genetic perturbation of an essential gene. In short, they investigated the initial physiological response to this perturbation then also performed an adaptive laboratory evolution (ALE) experiment to shed light on the possible evolutionary mechanisms that could mitigate the harmful effects. Briefly, they created conditional alleles of a nuclear pore protein complex member, and as a consequence generated truncated proteins with low abundance. They found that functionally related genes got upregulated as a physiological response, and this paved the way for the emergence of genomic mutations that compensated the initial fitness defect. This could potentially mean that inhibiting these early transcriptional changes during targeted drug therapy could be a useful strategy to limit the development of resistance towards the targeting agent.

Major comments:

I felt that the manuscript would require some additional analyses and extension of the text to fill in some gaps in order to provide some further support for their conclusions and also to increase clarity. See my detailed comments below.

1.The authors selected several nuclear pore proteins, all essential, and created conditional alleles. They also targeted a non-essential gene as a control. Maybe I am missing something or I have limited knowledge in working with human cell lines, but it was strange to read that even after targeting a non-essential gene, a 40 % drop in clone viability was observed according to Fig1C. Could this be a simple consequence of the potential off-target effect of CRISPR or did other unrelated factors affect viability? May it be the case that the transcriptome profile of the ancestor lines partly reflect a physiological response to potential off-target effects? Have the authors considered such possibilities? On a related note, Fig 1C shows relative viability, so basically, we don't have any information for the viability of cells transfected with a vector that lacks a gRNA.

R: We thank the Reviewer for giving us the possibility of explaining this phenomenon. Cell death after genome editing is a well-known side effect caused by Cas9 that is especially observed when multiple gRNAs are used concomitantly, a situation that generates several simultaneous independent DNA damage events, activates p53 and leads to cell death (Haapaniemi et al., 2018 Nat. Med.; Schiroli et al., 2019 Cell Stem Cell). Accordingly, as shown in Figure EV1D, there is no significant difference in the viability between EV and CHMP1B edited HAP1 p53 null cells. We therefore hypothesize that the 40% reduction is due to p53 activation and not due to secondary off target mutations. To further corroborate out hypothesis, we note that: 1- the Cas9 enzyme that was used in the current study is a high-fidelity endonuclease (Havlicek et al., 2017 Mol. Ther.); 2- gRNAs targeting CHMP1B had no predicted possible off-targets in any essential genes. We therefore think that the 40% drop in viability observed while editing CHMP1B in HAP1 cells is a consequence of the activation of p53 in response to the Cas9-induced DNA damage.

Unnormalized counts could be misleading as they do not report the frequency of viability. We consistently observe in our lab the viability of single cell HAP1 clones after transfection to be ~30%, regardless of the backbone. While this might look low, it is expected given the stress related to transfection, single-cell sorting and the amplification step from a single cell.

2.The Authors have used 6 clones with perturbed NUP58 gene for further in-depth analysis. All of the clones showed a significant fitness decrease based on Fig 2C with a somewhat similar effect size for most of them. However, the cargo efficacy experiment demonstrated that some of the clones had WT-like nuclear transport. The Authors proposed a possible explanation for that in the form of rapid evolution during the transfection/selection steps of the reporter construct. This could have been possibly tested by re-measuring the fitness of the clones carrying the reporter construct and showing WT-like nuclear transport and thereby demonstrating that they no longer have any large fitness defect. By the way, exactly how many generations are we talking about right here during this rapid evolution? Would we expect genetic or transcriptional changes during this presumably short period of time?

R: In the experiment showed in Figure 2D the 6 clones were transduced at P1 with the NLS-3XmCherry construct. Cell transfection is a stressful step for mammalian cells and leads to cell death and selection for fitter cells. We therefore think that the transfection step selected for the best adaptants that had less impairment. Therefore, to experimentally test that NUP58 mutant clones have impaired fitness and ability to shuttle cargos between the nucleoplasm and the cytoplasm, we first stably expressed the NLS-3XmCherry reporter in HAP1 cell line and then generated fresh NUP58 mutant clones by CRIPSR/Cas9. These clones were immediately analyzed for their fitness (measured by quantitative growth rate), nuclear import ability and presence of hypomorphic NUP58 mutations. As we predicted, all analyzed unpassaged early-derived NUP58 clones showing decreased NUP58 expression had concomitant impairment in nuclear import and fitness (new Figure 2B-C). We also collected a WT clone, which, as expected, did not show growth or nuclear import defects (Figure 2 clone A). These results are in agreement with our hypothesis that the slow growth phenotype of NUP58 mutant clones is specifically driven by inactivating NUP58 mutations and not by CRISPR/Cas9 aspecific

secondary targets. These results are also in agreement with our initial hypothesis that absence of nuclear import in some NUP58 clones (new Figure 3C) was due to cellular adaptation that occurred during the process of transfection and selection to introduce the NLS-3XmCherry reporter in the mutant clones.

3.According to Fig EV2A, the total NUP58 read count (from the RNA-sequencing dataset) was decreased in all clones (interpreted as destabilized mRNA), however a big variation was apparent across the clones (ranging from ~0.4 to ~0.8 relative expression), an observation that was not visible in Fig EV2B, where protein levels were plotted. It would be helpful to have an interpretation for this difference in the manuscript (e.g. post-transcriptional regulation?).

R: In this work we performed MS2-based proteomics, for which the absolute quantification of fold-change might be skewed due to ratio compression (Ting et al., 2011 Nat Met). Although MS2 is accurate to determine differences in abundance between different biological conditions, the real ratios might be higher than those identified and therefore a direct comparison between RNAseq and MS2 datasets might be difficult to make.

4.It might be informative to investigate if there is any correlation between expression level/protein level/transport efficacy and fitness across the lines (both ancestor and evolved).

R: We understand this would be a very interesting analysis. However, six mutant clones represent a too small sample to perform a robust correlation analysis, as the data points are not enough to draw any meaningful conclusion; six cell lines are quite heterogeneous, i.e. not spread in the smooth spectrum of a continuous fitness scale.

5.Expression analysis of the different clones revealed major differences in the transcriptome profile of different clones. For example, clone 3 and clone 5 display a totally different response to the perturbation of NUP58, also suggesting different off-target effects across the clones (ie. see major comment 1). The authors might consider trying to cluster the clones according to their profile, in addition to Fig App S3, where the transcripts were clustered. By doing that, clones with similar profiles would be more visible. Also by having that we might be able to better understand the phenotypic differences across the lines.

R: As discussed in response to point 1 and shown by the new exome sequencing data analysis in Appendix Figure S3A, we respectfully disagree the clones carry several off-site targets that can affect the clone phenotypes. Also, based on Figure EV3A, we respectfully disagree with the statement that C3 and C5 show a "totally different response". While there are some transcriptome differences, these could be easily explained by difference in growth rates, etc. Ultimately, we were looking for adaptive mutations. By analyzing all clones together, we found karyopherins to be upregulated and as shown in new Figure 5D their upregulation is sufficient to increase viability of freshly generated NUP58 mutants.

6.The Authors report in the manuscript that NPC components are generally downregulated. Maybe I am missing something, but I failed to see that in Fig EV3A, where mRNA levels were

plotted in each clone relative to the wild-type control line. Out of the NPC components, only the NUP54 are marked besides NUP58 (ie. the perturbed gene), and it does not seem to reach either the fold-change or the p-value cutoff, at least in the case of transcript levels. The protein level of NUP54 seems to be more likely in accordance with the text (and with Fig4), although some of the clones still don't seem to have a lower NUP54 protein level. The same applies to the upregulated karyopherins that do not seem to be upregulated in all of the clones (even at protein level). It would be helpful if the Authors could elaborate on this, and explain the rationale for the calculation of the average instead of the median value of the clones on Fig 4A-B, which would have been less sensitive to an outlier clone. Although I am still not sure if calculating summary statistics (mean or median of 6 clones) of expression levels is the right way to make any inferences on the response in general as the clones have different fitness/cargo efficacy. I also recommend to display these functional groups with a different color (or shape) on Fig EV3A-B for clarity. Alternatively, make another plot that shows only these functional categories. Do we really see them as being significantly up/downregulated? Do we have a significant enrichment of these functional categories among the up/downregulated genes? This can be easily tested by performing Fisher's exact test to probe whether being/not being in the functional category shows any association with being/not being in the set of up/downregulated genes.

R: We thank the Reviewer for giving us the opportunity to explain this further. As shown in Figure EV3A and EV3B there is a discrepancy between the mRNA and the protein levels of NUP54. We think this is a consequence of the selective degradation of NUP54 at the protein level. Indeed, it is well known that proteins of NPC subunits are often degraded if their interacting partner(s) is absent or the stoichiometric ratio of the complex is otherwise out of balance (Boehmer et al., Proc Natl Acad Sci U S A, 2003). So in this case, while NUP54 mRNA abundance is as in control cells, NUP54 protein is unstable and degraded as it cannot interact with its binding partner NUP58. We have now added this explanation to the figure legend of EV3A and B.

Also, in response to the reviewer's comment, we have reanalyzed the RNAseq and MS datasets to calculate the median of the gene and protein changes across the 6 clones. As shown in new Figure 5A and B, there is no quantitative difference between the average (previous Figure 4A-B) and the median. Also, we respectfully disagree with the reviewer that, based on their nuclear import efficiency, the 6 clones are different and that therefore the calculation of the summary statistics of all clones is meaningless. As discussed above, new Figure 3C showed a differential import ability in between the 6 clones. This difference is most likely due to the transfection, selection and evolution process the clones had to go through to insert the reporter gene. Indeed, as shown in new Figure 2C, all freshly analyzed NUP58 mutant clones show a similar level of impairment in respect to wild-type. Lastly, as now shown in new Figure 5C and 5D, the overexpression of KPNB1 and KPNA4, which accordingly to our analysis are upregulated in the 6 clones, is sufficient to increase the viability of freshly generated NUP58 mutant clones, further corroborating the validity of our analysis.

7.Upregulation of karyopherins (transport receptors) seems to be an early adaptive response to the perturbed NUP58 function. Would it be possible to test this hypothesis by artificial overexpression of such a protein? I also wonder if there is an expression limit for a nuclear membrane protein without having any toxic effect (ie. dosage sensitivity). The Authors might

also consider finding some further support for their hypothesis by looking for synthetic sick/lethal interaction between the yeast homologs of nuclear pore proteins and karyopherins based on previous genetic interaction screens in baker's yeast.

R: This is a great point. As shown in new Figure 5A-5B, unpassaged early-derived NUP58 mutant clones upregulated the mRNA and protein expression of TNPO1-TNPO2-KPNB1-KPNA4 karyopherins. To test if karyopherins upregulation is sufficient to sustain early adaptation in response to NUP58 mutations, we needed to overexpress karyopherins. This experimental procedure was not trivial as, as mentioned by this Reviewer, the overexpression of certain karyopherins, such as TNPO1, is toxic in both mammalian and bacterial cells. After trying different strategies, we successfully integrated KPNB1-KPNA4 under doxycycline-inducible promoter in a wild-type HAP1 cell line. As shown in new Figure 5D, induction of KPNB1-KPNA4 karyopherin overexpression significantly increase the viability of freshly generated NUP58 mutant clones. We also noted that there is a slight increase in the viability of NUP58 single cell-derived clones in absence of doxycycline treated. This is expected as the doxycycline expression system is documented to be leaky with reported basal expression of the transgenes in absence of doxycycline (Meyer-Ficca et al., 2004 Anal. Biochem., Pham et al., 2008 Biothechniques). This result is in agreement with our hypothesis that early adaptation to NUP58 inactivating mutations is driven by phenotypic plasticity and specifically by a transient transcriptional upregulation of the karyopherins.

In terms of genetic interaction, this is a great point. We found several negative and positive genetic interactions present in yeasts between karyopherin (KAP120), karyopherin beta (KAP123 and KAP95), transportin (KAP104) with several NPC components (Costanzo et al, 2016 Science, Wilmes GM et al., 2008 Mol. Cell, Kuzmin E, et al. 2018 Science and Ryan KJ et al., 2007 Mol. Biol. Cell). Also, as shown in Koh et al., 2015 Cell, the C-terminal FG domain of NUP58-NUP54 can 'sense' the concentration of karyopherins and undergoes a conformational change that depends on the local concentration of karyopherins. Specifically, in presence of karyopherins, NUP58-NUP54 FG-repeats switch from a constricted to a dilated conformation to allow the passage of the nuclear transporter proteins through the pore channel. In the presence of high local karyopherins concentration, the pore dilation is such that karyopherins can diffuse through the pore in a more FG-independent manner. These findings are in accordance with our observation that a modest upregulation of karyopherins could facilitate the active transport of cargoes through the pore in mutants of NUP58 lacking the FG repeats. This session has now been added to the discussion.

8.The evolutionary experiment lasted for 20 passages, ie. 40 generations, which seems to be a quite short evolutionary experiment, compared to those conducted in single-celled microorganisms, where hundreds of generations are reached. Although I am not an expert in evolutionary studies in human cell lines, this is all the more intriguing if we consider that the mutational supply depends on the mutational rate and population size, both of which I would expect to be lower in human cells compared to bacteria/yeast. Since we don't have any information on these factors in the current setup, a solid explanation and a more detailed methods section (currently there is no such) for the evolutionary experiment would be required.

R: While, as noted by the Reviewer, there are many adaptive evolution experiments performed in yeasts or bacteria, this is, to our knowledge, the first experiment published using human cells. So, while we agree with the Reviewer that the number of passages is relative low and the population size is small when compared to yeast or bacterial evolution experiments, we decided to halt the passaging as mutant cells reached a plateau in the growth rate recovery (Figure EV4), suggesting clones have reached a (at least local) fitness peak. It is conceivable that further passaging would lead to further adaptation. However, given the fact that fitness remained stable for the last passages, we reasoned that it would take several thousand of passages before being able to potentially capture that event, making such experiment difficult to perform in mammalian cells as it would possibly require one year or more. As stated in the response to Reviewer #1 "minor point" 1, we added a more detailed explanation of the number of generations and population size in the methods section.

9.Fig EV4A shows the fitness of the 6 clones before and after evolution. I made several observations by looking at this figure. First, C2 seems to be an outlier, as it has no significant fitness cost at P1 compared to the WT. Could this be due to the karyopherin overexpression? Does this clone have a higher expression level than the rest of the clones? Second, clone 4 did not improve its fitness, as a matter of fact, it seems that there is a fitness decline at the end of the experiment. Is there any explanation for that? Could it be due to lower initial upregulation of karyopherins? Third, it is hard to tell, whether there is any main difference in the relative fitness improvement among the rest of the clones. This can be easily tested if the Authors normalize the fitness of each passage with the first one (or maybe even to the passage 0, ie fitness displayed on Fig 2C?).

R: We have extensively reviewed the mRNA, MS and growth rate datasets of clone C2. However, we are currently unable to explain the molecular underpinning of its fitness at P1 in Figure EV4A.
We, on the other hand, found a plausible explanation for the behavior of C4 clone during the evolution experiment. Indeed, while in all clones NUP58 levels increase upon passaging, we noted that C4 has the least increase as detected by RNAseq and MS (Figure EV3). This was also confirmed by western blot analysis of NUP58 at P1 and P19 of all clones. Indeed, as shown in new Figure Appendix S6A, C4 is the only clone that does not increase the expression of NUP58 mutant protein upon passaging. We therefore hypothesized that clone C4 is unable to restore fitness during the evolution experiment because it fails to upregulate the expression of NUP58 mutant protein. Regarding the fitness decline of clone C4 reported by the Reviewer, we noted that there is not a statistically significant decrease in the fitness of this clone, suggesting that the growth rate does not improve.
Lastly, we are not able to show the relative fitness as we only performed one replicate of the evolution experiment.

10.The Authors chose C5 for in-depth analysis, even though they do not provide any criteria for that. They found that the NPC was stabilized, demonstrated by increased efficacy of nuclear transport (Fig5B) and increased NUP58 protein level (Fig5D). As a possible mechanism, they found a strong focal amplification (demonstrated by FISH) of the NUP58 instead of chromosome aneuploidy. However, this was not reflected in protein level, as it still seems to have a

significantly lower protein level than the non-truncated NUP58 level in the WT (Fig5D), although the discussion claims it otherwise: "restoration of mutant protein expression to wild-type levels correlated with a significant fitness gain". Nevertheless, the results suggest there is a large contribution of posttranscriptional regulation that keeps the mutant protein level under the wild-type. This is quite intriguing as without such a regulation the strong amplification of NUP58 would also imply a stoichiometric imbalance among the members of the NUP complex which should subsequently result in a fitness loss as well. I would appreciate any comment on this issue.

R: Clone 5 was initially chosen as it concomitantly displayed impaired fitness and nuclear import activity. Moreover, C5 NUP58 carries mutations in both exon 4 and 13, making it one of the most crippled allele. This comment has now been added to the text.

As the reviewer noted, while the protein abundance is not restored to wild-type levels, mutant NUP58 levels increase upon passaging. We agree with the Reviewer that possible post-transcriptional events could be occurring, however we prefer the view that the hypomorphic allele is transcriptionally unstable and therefore even if the allele is overexpressed, the protein levels are not restored to control levels. We have now added this comment to the text and tone down the statement of the restoration to wild-type levels.

Minor comments:

1.The manuscript is well written. The figures are well designed and provide clear messages.

R: We thank the reviewer for his/her positive comments on the text and figures.

2.Fig 5B is not referred to in the text.

R: We are sorry for this missing reference. We amended the text to refer Figure 5B.

3.The method(s) to correct the p-values for multiple testing is not mentioned in the text, please add it accordingly.

R: We added the p-value method used to the figure legends for all instance a p-value was used.

4.Prior work(s) that investigated the compensability of essential gene perturbations using baker's yeast as a model should be cited in this manuscript.

R: We agree with the Reviewer. We will add to the discussion previous adaptive evolution experiments using budding yeast, for instance Liu et al, Cell 2015.

Reviewer #3 (Significance (Required)):

The subject and the general plan of the present study are well chosen. It was stimulating to read an ALE experiment that used a human cell line instead of the commonly used single-celled microorganisms like yeast and bacteria. I believe that such a work could potentially pique the interest of anyone in the broad field of evolutionary biology and potentially also beyond that. Similar works like this current study could help in better understanding whether the general principles we learned about compensatory mechanisms in the simpler model organisms can also be applied across different kingdoms of Eukaryotes. Since I work in the field of yeast biology and I am not an expert of human-related techniques/protein techniques (eg. Western blot) I might found some of the methods/results section inappropriate. If they follow established protocols/concepts, please ignore my corresponding comments.

R: We really would like to thank the Reviewer for his/her support on this point. As he/she pointed out, our work is the first to dissect adaptive mechanisms in human cells. This is of paramount importance to understand fundamental and general principle of evolution across eukaryotes.

Dear Dr. Targa,

Thank you for submitting your revised manuscript, we have now received the reports from the three initial referees (see comments below). I am pleased to say that they overall find that their comments have been satisfactorily addressed and in principle now support publication. However, referee #2 does find that one of her/his initial issues is not yet fully resolved and this should be addressed in the final version of the manuscript. Please also provide a brief point-by-point response to these comments when resubmitting the revised manuscript. In addition, in this version, I would also ask you to address a number of editorial issues that are listed in detail below. Once these remaining issues are resolved, we will be happy to formally accept the manuscript for publication.

Referee #1:

The authors have properly addressed my previous concerns, and I have no further comments.

Referee #2:

The revised manuscript and the particular replies to the concerns raised previously are satisfactory. The extra experiments were carried out properly, and they helped to improve the state of the manuscript significantly. I, therefore, find the paper worthy of publication in EMBO Journal

There is only one minor thing that was probably not clear in my previous comments. It would have been useful to have a Supplementary Fig that shows the relative fitness improvements of the clones after the evolutionary experiment compared to the initial fitness. At a first glance, it seems that clones with smaller fitness defects at the beginning show smaller fitness improvement which would be in good accordance with the declining adaptability concept (e.g. PMID: 25815007).

This was my particular question:

quote:
"9.Fig EV4A shows the fitness of the 6 clones before and after evolution. I made several observations by looking at this figure.
...
Third, it is hard to tell, whether there is any main difference in the relative fitness improvement among the rest of the clones. This can be easily tested if the Authors normalize the fitness of each passage with the first one (or maybe even to the passage 0, ie fitness displayed on Fig 2C?)."

The Authors might have misunderstood as they replied as follows:
quote:
"Lastly, we are not able to show the relative fitness as we only performed one replicate of the evolution experiment."

On the particular figure, I can see multiple data points for each clone (are they biological or technical replicates?). If they normalize every data point with the mean of the corresponding P1 (both for WT and C1-C6, separately) then they can plot the relative fitness improvement for each genotype, as the P1 of a given genotype will have a mean fitness of 1. On a side note, the WT also seems to have a larger fitness at P19, which seems to be non-significant, but this is something that would be probably desirable to highlight and thereby refute potential adaptation to the experimental conditions in the WT.

Referee #3:

The authors have addressed all points raised, I have no further comments to add.

Dear Dr. Boehm,

We apologize for not having initially understood Reviewer#2 suggestion. We have now added a new Appendix Figure S5A to the manuscript to illustrate the relative fitness improvement of NUP58 mutant clones. We also added a comment in the text highlighting that clones with smaller fitness defects at P1 showed smaller fitness improvement during passaging. Lastly we added that HAP1 WT showed a non-significant fitness gain upon passaging, thereby suggesting that the observed fitness increases in the clones are not due to adaptation to the experimental conditions (lines 225-230).

2nd Revision - Final Decision 23rd Aug 2021

Thank you again for submitting the final revised version of your manuscript. Please excuse the delay in communicating the decision to you, which is due to slower responses over the summer holiday period as well as my own absence from the office. You will likely hear from me again as we finalize the files for transfer to the publisher, but for now, I am pleased to inform you that we have formally accepted your manuscript for publication in The EMBO Journal.

EMBO PRESS

YOU MUST COMPLETE ALL CELLS WITH A PINK BACKGROUND ↓

PLEASE NOTE THAT THIS CHECKLIST WILL BE PUBLISHED ALONGSIDE YOUR PAPER

Corresponding Author Name: Giulia Rancati
Journal Submitted to: The EMBO Journal
Manuscript Number: EMBOJ-2021-107839R

Reporting Checklist For Life Sciences Articles (Rev. June 2017)

This checklist is used to ensure good reporting standards and to improve the reproducibility of published results. These guidelines are consistent with the Principles and Guidelines for Reporting Preclinical Research issued by the NIH in 2014. Please follow the journal's authorship guidelines in preparing your manuscript.

A- Figures
1. Data
The data shown in figures should satisfy the following conditions:

- the data were obtained and processed according to the field's best practice and are presented to reflect the results of the experiments in an accurate and unbiased manner.
- figure panels include only data points, measurements or observations that can be compared to each other in a scientifically meaningful way.
- graphs include clearly labeled error bars for independent experiments and sample sizes. Unless justified, error bars should not be shown for technical replicates.
- if n< 5, the individual data points from each experiment should be plotted and any statistical test employed should be justified
- Source Data should be included to report the data underlying graphs. Please follow the guidelines set out in the author ship guidelines on Data Presentation.

2. Captions

Each figure caption should contain the following information, for each panel where they are relevant:

- a specification of the experimental system investigated (eg cell line, species name).
- the assay(s) and method(s) used to carry out the reported observations and measurements
- an explicit mention of the biological and chemical entity(ies) that are being measured.
- an explicit mention of the biological and chemical entity(ies) that are altered/varied/perturbed in a controlled manner.

- the exact sample size (n) for each experimental group/condition, given as a number, not a range;
- a description of the sample collection allowing the reader to understand whether the samples represent technical or biological replicates (including how many animals, litters, cultures, etc.).
- a statement of how many times the experiment shown was independently replicated in the laboratory.
- definitions of statistical methods and measures:
  - common tests, such as t-test (please specify whether paired vs. unpaired), simple χ2 tests, Wilcoxon and Mann-Whitney tests, can be unambiguously identified by name only, but more complex techniques should be described in the methods section;
  - are tests one-sided or two-sided?
  - are there adjustments for multiple comparisons?
  - exact statistical test results, e.g., P values = x but not P values < x;
  - definition of 'center values' as median or average;
  - definition of error bars as s.d. or s.e.m.

Any descriptions too long for the figure legend should be included in the methods section and/or with the source data.

In the pink boxes below, please ensure that the answers to the following questions are reported in the manuscript itself. Every question should be answered. If the question is not relevant to your research, please write NA (non applicable). We encourage you to include a specific subsection in the methods section for statistics, reagents, animal models and human subjects.

USEFUL LINKS FOR COMPLETING THIS FORM

http://www.antibodypedia.com
http://1degreebio.org
http://www.equator-network.org/reporting-guidelines/improving-bioscience-research-repor
http://grants.nih.gov/grants/olaw/olaw.htm
http://www.mrc.ac.uk/Ourresearch/Ethicsresearchguidance/Useofanimals/index.htm
http://ClinicalTrials.gov
http://www.consort-statement.org
http://www.consort-statement.org/checklists/view/32-consort/66-title
http://www.equator-network.org/reporting-guidelines/reporting-recommendations-for-tumo
http://datadryad.org
http://figshare.com
http://www.ncbi.nlm.nih.gov/gap
http://www.ebi.ac.uk/ega
http://biomodels.net/
http://biomodels.net/miriam/
http://jjj.biochem.sun.ac.za
http://oba.od.nih.gov/biosecurity/biosecurity_documents.html
http://www.selectagents.gov/

B- Statistics and general methods

Please fill out these boxes ↓ (Do not worry if you cannot see all your text once you press return)

1.a. How was the sample size chosen to ensure adequate power to detect a pre-specified effect size?	Information on sample size for each independent experiment is provided under respective figure legends and materials and methods section.
1.b. For animal studies, include a statement about sample size estimate even if no statistical methods were used.	NA
2. Describe inclusion/exclusion criteria if samples or animals were excluded from the analysis. Were the criteria pre-established?	If any samples were not included in the analysis, exclusion criteria are described within the manuscript text.
3. Were any steps taken to minimize the effects of subjective bias when allocating animals/samples to treatment (e.g. randomization procedure)? If yes, please describe.	NA
For animal studies, include a statement about randomization even if no randomization was used.	NA
4.a. Were any steps taken to minimize the effects of subjective bias during group allocation or/and when assessing results (e.g. blinding of the investigator)? If yes please describe.	NA
4.b. For animal studies, include a statement about blinding even if no blinding was done	NA
5. For every figure, are statistical tests justified as appropriate?	Description for statistics test for each figure is provided under respective figure legends and Data Quantification section
Do the data meet the assumptions of the tests (e.g., normal distribution)? Describe any methods used to assess it.	Data meet assumptions of statistical tests used as described in repsective figure legends.
Is there an estimate of variation within each group of data?	Estimates of variation are provided for each group of data provided under respective figure legends and materials and methods section.

Is the variance similar between the groups that are being statistically compared?	Groups being statistically compared have appropriate variance similarities for the respective statistical tests used as described in the accompanying figure legends and materials and methods section.

C- Reagents

6. To show that antibodies were profiled for use in the system under study (assay and species), provide a citation, catalog number and/or clone number, supplementary information or reference to an antibody validation profile. e.g., Antibodypedia (see link list at top right), 1DegreeBio (see link list at top right).	Antibodies used in this study include Ab1 NUP58 customized by GL Biochem (Appendix Table S2), Ab2 NUP58 customized by GenScript (Appendix Table S2), anti-CHMP1B (Santa Cruz, sc514013), anti-p53 (Thermo, MA512571), anti-NUP153 (abcam QE5 ab24700), anti-NUP85 customized by GL Biochem (Appendix Table S2), anti-actin (MA5-15739).
7. Identify the source of cell lines and report if they were recently authenticated (e.g., by STR profiling) and tested for mycoplasma contamination.	HAP1 cell line was purchased from Horizon Discovery (C859). HCT116 and 293T cells were obtained from the American Type Culture Collection (Rockville, MD). All lines were tested for mycoplasma contamination regularly and confirmed negative. HAP1, HCT116, and HEK293T cells were cultured under standard conditions as described in M&M)

* for all hyperlinks, please see the table at the top right of the document

D- Animal Models

8. Report species, strain, gender, age of animals and genetic modification status where applicable. Please detail housing and husbandry conditions and the source of animals.	NA
9. For experiments involving live vertebrates, include a statement of compliance with ethical regulations and identify the committee(s) approving the experiments.	NA
10. We recommend consulting the ARRIVE guidelines (see link list at top right) (PLoS Biol. 8(6), e1000412, 2010) to ensure that other relevant aspects of animal studies are adequately reported. See author guidelines, under 'Reporting Guidelines'. See also: NIH (see link list at top right) and MRC (see link list at top right) recommendations. Please confirm compliance.	NA

E- Human Subjects

11. Identify the committee(s) approving the study protocol.	NA
12. Include a statement confirming that informed consent was obtained from all subjects and that the experiments conformed to the principles set out in the WMA Declaration of Helsinki and the Department of Health and Human Services Belmont Report.	NA
13. For publication of patient photos, include a statement confirming that consent to publish was obtained.	NA
14. Report any restrictions on the availability (and/or on the use) of human data or samples.	NA
15. Report the clinical trial registration number (at ClinicalTrials.gov or equivalent), where applicable.	NA
16. For phase II and III randomized controlled trials, please refer to the CONSORT flow diagram (see link list at top right) and submit the CONSORT checklist (see link list at top right) with your submission. See author guidelines, under 'Reporting Guidelines'. Please confirm you have submitted this list.	NA
17. For tumor marker prognostic studies, we recommend that you follow the REMARK reporting guidelines (see link list at top right). See author guidelines, under 'Reporting Guidelines'. Please confirm you have followed these guidelines.	NA

F- Data Accessibility

18: Provide a "Data Availability" section at the end of the Materials & Methods, listing the accession codes for data generated in this study and deposited in a public database (e.g. RNA-Seq data: Gene Expression Omnibus GSE39462, Proteomics data: PRIDE PXD000208 etc.) Please refer to our author guidelines for 'Data Deposition'.<br><br>Data deposition in a public repository is mandatory for:<br>a. Protein, DNA and RNA sequences<br>b. Macromolecular structures<br>c. Crystallographic data for small molecules<br>d. Functional genomics data<br>e. Proteomics and molecular interactions	The mass spectrometry proteomics data have been deposited to the ProteomeXchange Consortium via the PRIDE partner repository with the dataset identifier PXD022255. The RNAseq data presented have been deposited in the Gene Expression Omnibus repository (https://www.ncbi.nlm.nih.gov/geo/) with the accession number GSE161061. The exome sequencing data have been deposited in the NCBI Sequence Read Archive (SRA) with the BioProject accession number PRJNA680690.
19. Deposition is strongly recommended for any datasets that are central and integral to the study; please consider the journal's data policy. If no structured public repository exists for a given data type, we encourage the provision of datasets in the manuscript as a Supplementary Document (see author guidelines under 'Expanded View' or in unstructured repositories such as Dryad (see link list at top right) or Figshare (see link list at top right).	See (18) above
20. Access to human clinical and genomic datasets should be provided with as few restrictions as possible while respecting ethical obligations to the patients and relevant medical and legal issues. If practically possible and compatible with the individual consent agreement used in the study, such data should be deposited in one of the major public access-controlled repositories such as dbGAP (see link list at top right) or EGA (see link list at top right).	NA
21. Computational models that are central and integral to a study should be shared without restrictions and provided in a machine-readable form. The relevant accession numbers or links should be provided. When possible, standardized format (SBML, CellML) should be used instead of scripts (e.g. MATLAB). Authors are strongly encouraged to follow the MIRIAM guidelines (see link list at top right) and deposit their model in a public database such as Biomodels (see link list at top right) or JWS Online (see link list at top right). If computer source code is provided with the paper, it should be deposited in a public repository or included in supplementary information.	NA

G- Dual use research of concern

22. Could your study fall under dual use research restrictions? Please check biosecurity documents (see link list at top right) and list of select agents and toxins (APHIS/CDC) (see link list at top right). According to our biosecurity guidelines, provide a statement only if it could.	No- NA
